# Short-term exposure to ultrafine and fine particulate matter with multipollutant modelling on heart rate variability among seniors and children from the CorPuScula (coronary, pulmonary, sanguis) longitudinal study in Germany

**DOI:** 10.3389/fepid.2023.1278506

**Published:** 2023-11-08

**Authors:** Pascale Haddad, Katherine Ogurtsova, Sarah Lucht, Lina Glaubitz, Peter Höppe, Dennis Nowak, Peter Angerer, Barbara Hoffmann

**Affiliations:** ^1^ Institute for Occupational Social and Environmental Medicine, Centre for Health and Society, Medical Faculty and University Hospital Düsseldorf, Heinrich Heine University Düsseldorf, Düsseldorf, Germany; ^2^ Real-World Evidence & Insights, Cardinal Health, Dublin, OH, United States; ^3^ Institute and Outpatient Clinic for Occupational, Social and Environmental Medicine, Ludwig-Maximilians-University Munich, Munich, Germany

**Keywords:** ultrafine particles, heart rate variability, co-pollutants, children, seniors, sympathetic and para-sympathetic nervous systems

## Abstract

**Background:**

Short-term exposure particulate matter with a diameter of 10 µm or less (PM_10_) and fine particulate matter (PM_2.5_) has been associated with heart rate variability (HRV), but exposure to ultrafine particles (UFP) has been less well examined. We investigated the associations between the HRV outcomes and short-term exposure to UFP, PM_10_ and PM_2.5_ among school-aged children and seniors.

**Methods:**

CorPuScula (Coronary, Pulmonary and Sanguis) is a longitudinal, repeated-measure panel study conducted in 2000–2002 in Munich, Germany including 52 seniors (58–94 years old) with 899 observations and 50 children (6–10 years old) with 925 observations. A 10-min resting electrocardiogram was performed to assess resting HRV outcomes [Standard Deviation of Normal to Normal Intervals (SDNN), Root Mean Square of Successive Differences between Normal Heartbeats (RMSSD), Low Frequency power (LF), High Frequency power (HF), ration between low and high frequency (LF/HF)]. UFP and PM exposures were measured near the care home and school yard for seniors and children, respectively. Mean exposures during the day of examination (9–21 h) as well as 3-h, 12-h, 24-h, one-day, and two-day lags were assessed. Linear mixed-effect models were used to investigate the associations between short-term air pollution and HRV outcomes separately in children and seniors. The models were adjusted for sex, age, weather conditions (temperature, precipitation, and water vapor pressure), BMI, lifestyle and medical information. Two and multipollutant models adjusted for NO_2_ and O_3_ were performed.

**Results:**

Among seniors, we observed increases in SDNN, LF, HF and LF/HF ratio after short-term exposure to UFP (hourly and daily lags) in contrast to decreases in SDNN and RMSSD after exposure to PM_10_. Associations were generally robust to two- and multipollutant adjustment. Among children, we observed increases of the LF/HF ratio after short-term exposures to UFP at lags 12 and 24 h. In contrast, we observed decreases of the ratio after exposure to PM_2.5_ and PM_10_. Results were largely unchanged for multipollutant modelling, however we found a more pronounced increase in SDNN and LF/HF (UFP lag 12 and 24 h) after adjusting for NO_2_.

**Conclusions:**

Overall, among seniors, we observed associations of UFP and PM_10_ exposure with sympathetic responses of the ANS, which play an important role in sudden heart attacks or arrhythmia. Among children we found more inconsistent associations between UFP and a delayed increase in HRV. Adjusting for co-pollutants including NO_2_ and O_3_ yielded robust results.

## 1. Introduction

Heart rate (HR) is the number of heart beats per given time period, conventionally given per minute. Heart rate variability (HRV) is the fluctuation in the time intervals between successive heart beats (1). HRV is a non-invasive dynamic metric and assesses the activity of the autonomic nervous system (ANS), including both the sympathetic and parasympathetic nervous systems (SNS and PNS, respectively). Importantly, the ANS has a well-established role as an indicator of cardiovascular disease (CVD) risk among adults (2–5). The ANS controls involuntary actions like HR, body temperature, digestion, perspiration, and the widening or narrowing of blood vessels (1). In the past decades, several studies have attributed imbalance in the ANS to some CVD clinical conditions such as sudden death, coronary artery disease, or heart failure (6). The ANS regulates the HR and blood pressure in the short-term to cope with everyday situations (7). A higher HRV indicates better general health (8), whereas a reduced HRV has been reported among children with Attention Deficit/Hyperactivity Disorder (9) or overweight and obesity (10). Moreover, among adults, a reduced HRV is associated with the development of cardio-metabolic diseases such as diabetes (11).

The literature shows that ambient air pollution, and more specifically the exposure to fine particulate matter (PM), is a major threat to older people with a CVD history and causally contributes to CVD morbidity and mortality in general (12–17). However, fewer studies have assessed the effects of ultrafine particles (UFP) with an aerodynamic diameter of ≤100 nm on CVD. UFPs are of great interest since they have the ability—due to their small size—to reach the pulmonary alveoli, from where they can pass into the circulatory system and reach all organs (18). Specifically, the potential systemic translocation of UFP can lead to endothelial and vascular dysfunction, hypertension, thrombosis, atherosclerosis and eventually might promote vasoconstriction in the coronary arteries, increasing the risk of stroke, coronary heart disease, heart failure and ultimately mortality (18–23). Moreover, UFPs are thought to be more toxic than larger PM and to have independent adverse health effects since their high reactivity surface area enables them to adsorb a large amount of toxic metals and organic compounds. These compounds generate oxidative stress that leads to inflammation and eventually increases risk for cardiovascular and respiratory diseases (24, 25).

It has been hypothesized that the inhaled UFP, PM_10_ and PM_2.5_, can activate the pulmonary reflexes that modify the autonomic control of cardiovascular function and potentially mediate an immediate response (within a few hours) leading to an autonomic imbalance that can be assessed by HRV outcomes (26). The latest review and meta-analysis on short-term UFP exposure, including 12 studies published up to June 2022, found decreases in some HRV outcomes within hours and up to days of exposure (22). Specific studies on short-term UFP exposure (hours to days) have found consistent decreases in HRV among adults but very little information is available for children (21, 27–29). Moreover, recent studies showed consistent decreases in HRV outcomes with short-term exposures to fine PM (20, 21). To our knowledge, only one study reported the same inverse association between PM and HRV in children (30). Moreover, the use of multipollutant modelling in this area is also scare. Previous work has investigated the effects of PM_2.5_ and ozone (31) on HRV, and some studies have looked at the adverse effects of two-pollutant models (UFP and PM_2.5_) on HRV (32–34). However, it is also important to look at the effect of NO_2_ since UFP and NO_2_ share traffic as their main source and might be correlated. Without adjusting for co-pollutants, it is challenging to attribute any association with HRV to UFP alone.

We therefore investigated the association of short-term exposure to UFP, PM_10_ and PM_2.5_ with HRV among elementary school children and seniors in a care-home in single, two and multi-pollutant models taking into account other co-pollutants (i.e., NO_2_, O_3_ and PM_2.5_).

## 2. Materials and methods

### 2.1. Study design and participants

CorPuScula (Cor for coronary, Pu for pulmonary and S for sanguis or blood) is a longitudinal repeated-measures panel study conducted in 2000–2002 by the Institute and Outpatient Clinic for Occupational, Social and Environmental Medicine, Ludwig-Maximilians-University (LMU) in Munich, Germany. The study included 52 seniors and 50 children.

#### 2.1.1. Seniors

The seniors were recruited via letter and an informational event at their care home in Munich (Wohnstift Augustinum Munich-North). Participation was voluntary and all those who consented to participate were invited to an initial one-hour long personal interview in the measurement van located outside their care home. Moreover, the participants filled in a standardized questionnaire and individual characteristics were collected including: age, height, weight, smoking habits, alcohol consumption, chronic illnesses, medication intake, and physician-diagnosed allergies. At the end of the initial interview, the participants gave written informed consent. Inclusion criteria for the seniors were as follows: (1) older than 55 years of age, (2) resident of the care home Wohnstift Augustinum Munich-North, and (3) time availability to complete the study over the upcoming year. The exclusion criteria included: (1) poor physical health that would prevent obtainment of clinical measurements, (2) current tobacco use, (3) presence of a pacemaker, and (4) intake of blood thinners (e.g., Marcumar). The participants were followed between May 2000 and July 2001 except during acute sickness. If possible, the appointments were scheduled at the same time of the day and the same day of the week to eliminate potential confounding from circadian rhythms or weekly effects. Routine examinations included the following: (1) a standardized questionnaire, (2) a bi-weekly 10-min electrocardiogram, (3) a bi-weekly blood sample, (4) blood-pressure measurement, and (5) lung-function measurements.

#### 2.1.2. Children

In the same way, the children were recruited at the Strehleranger elementary school in Munich-Neuperlach by sending a letter describing the study along with a response form to the parents/guardians. Participation was voluntary, and all parents/guardians who gave their consent were invited alongside their children to an initial one-hour long personal interview and filled in the initial questionnaire. For all participants, at least one parent/guardian was required to be present at the first interview. The only inclusion criterion was attendance at the Strehleranger elementary school. The only exclusion criterion was exposure to tobacco at home or in private life. The children were followed between September 2001 and July 2002 except during vacation, school holidays, or acute sickness. Similarly to the seniors and following the same strategy, the appointments were rescheduled if possible. Routine examinations included the same measurements as in the senior cohort except for the withdrawal of a blood sample. In this paper and for both groups, we will focus on the HRV outcomes only. A flow diagram reflecting all the targeted number of observations vs. the ones available and eventually used in the analyses can be found in Supplementary Figures S1A,B. This study was approved by the Ethics Committee of the Medical Faculty at LMU (No. 130/00).

### 2.2. Exposure assessment: air pollutants and meteorological parameters

UFPs (≤100 nm in diameter) were measured as particle number concentration (PNC, particles/cm^3^). The measurements for the seniors were carried out with a condensation particle counter (TSI 3022A, lower particle size detection limit >7 nm) which was located approximately 3 km away from the care home. For children, a particle counter (TSI 3025A, lower particle size detection limit >3 nm) was placed on the school grounds, which is located on the Strehleranger road. The calendar daytime mean concentrations and individual hourly and lagged exposures prior to the examinations were calculated from hourly means.

Particulate matter PM_10_ and PM_2.5_ concentrations were measured using a Low Volume Sampler (LVS3; Leckel brand) located in the park of the Wohnstift Augustinum and the courtyard of the Strehleranger elementary school for both the seniors and the children's cohorts, respectively. Measures of fine PM were collected for 24 h but only the concentrations between 9:30 am until 9:30 pm were used as these were the main times in which the participants were active outdoors. The daytime mean concentrations were then calculated and used in the analyses as lags 0, 1 and 2.

Exposure to other air pollutants and meteorological parameters (i.e., temperature, water vapor pressure, precipitation and wind speed) were collected at the measuring station Johanneskirchen (L8.12). The station was located 9 km north and 7 km south of the senior home and the school, respectively. We obtained air pollution data from the station for nitrogen dioxide (NO_2_, μg/m^3^) and ozone (O_3_, μg/m^3^). Meteorological variables for the senior cohort were obtained from the measuring station of the Meteorological Institute of the LMU in Munich (MIM), which is approximately 8 km from the care home. For the children's cohort, we obtained the meteorological data from the MIM's measuring station Theresienstrasse (approximately 7 km away). The meteorological data included hourly mean values for air temperature (°C), water vapor pressure (hPa), precipitation (mm) and wind speed (m/s). We calculated the corresponding daytime mean values for the measured air pollution intervals (9:30 am to 9:30 pm).

### 2.3. Outcomes: HRV measures

Every other week, HRV was measured using a long-term ECG monitoring system from the company Customed (Custo Flash 200). This monitor used 2 channels alongside 5 electrodes. Raw ECG data was processed and cleaned prior to HRV analyses by an experienced doctor. Quality control was performed for the 10-min measurement interval, and extra systoles were identified and removed from the dataset.

For data collection, participants lay relaxed on the bed for at least 5 min before and during the entire 10 min of the ECG examinations. They were instructed not to speak or move and to breathe calmly and evenly during the examination. We extracted the time-domain measures standard deviation of normal-to-normal intervals (SDNN) representing both the SNS and PNS activity, and the root mean square of successive differences between normal heartbeats (RMSSD) that is more strongly influenced by the PNS than SDNN. The frequency-domain measures yielded information about the total power in the heart's rhythm explained by the variability of the normal-to-normal intervals. These include the low-frequency power (LF, 0.04–0.15 Hz) influenced by both SNS and the PNS, the high-frequency power (HF, 0.15–0.4 Hz) reflecting the PNS, and the frequency ratio of LF/HF to estimate the balance between SNS and PNS activity.

### 2.4. Statistical analysis

We analyzed associations between UFP, PM_2.5_/PM_10_ and HRV outcomes using linear mixed-effects regression models separately in adults and children. The time variable in the models was the date of measurement. The models also included the personal identification number as random effect. The autocorrelation structure included the time covariate and the personal identification number as a grouping factor within the observations that were assumed correlated. We used both unweighted (homoscedastic) as well as weighted (heteroscedastic) models where we allowed variances to differ by person, with a control for time trend (35). The weights were calculated as a ratio of the stratum standard errors to the within-group standard error. We assessed the adequacy of the heteroscedastic fit by re-examining plots of the standardized residuals vs. the fitted values by stratum to check if the standardized residuals had the same variability in each stratum. Also, the fit was tested with the ANOVA method. 95% confidence intervals (CIs) and *p*-values were computed using a Wald *t*-distribution approximation.

For each pollutant (UFP, PM_10_ and PM_2.5_), we calculated lagged exposures including both single-day lags (i.e., lag 0, lag 1 and lag 2) as well as individual lags for PM_10_ (3, 12 and 24 h) and UFP (1, 3, 12 and 24 h).

The 5 outcomes (SDNN, RSMMD, LF, HF and LF/HF) were analyzed separately. In regression analyses, we first estimated the crude association between the lag periods for single pollutants with each outcome. Second, we estimated the same associations adjusting for possible confounders as found in previous studies (21, 36). To identify potential confounding and minimal sufficient adjustment sets, we constructed a directed acyclic graph (DAG) using the DAGitty program (37) separately for seniors and children. For seniors, the second model included age (years), sex (male/female), alcohol [did they drink on the day of the examination (yes/no), repeated measure], BMI (kg/m^2^), day of the week, precipitation, temperature, and water vapor pressure (see Supplementary Figure S2A). For children, the second model included age (years), sex (male/female), BMI (kg/m^2^), day of the week, precipitation, temperature, and water vapor pressure (see Supplementary Figure S2B). We did not adjust for multiple comparisons because the tests were highly correlated, thus violating the assumption of independence (see Supplementary Table S1 for correlations of outcome variables and the results section for correlations of exposure variables).

Meteorological variables (i.e., temperature, precipitation, and water vapor) were assessed for linearity using models without exposure variables, which resulted in modelling temperature and water vapor pressure with natural splines using 4 knots and precipitation with no splines. The best fitted lags for meteorological variables, which varied by outcome and group, were used (see Supplementary Table S1).

We then conducted two and multipollutant models by simultaneously adding the best fitted concentration (lag 0–2) of NO_2_ and O_3_ to the main models to examine the robustness of the effects after further controlling for the exposure (see Supplementary Table S2). Also, for models where the main exposure was UFP, we further adjusted for PM_2.5_ and vice versa.

We also ran further sensitivity analyses to assess the robustness of our results. For the seniors, we investigated the inclusion of the intake of heart medication (yes/no if they were prescribed a heart meds at the time) before every examination. We also repeated analyses including season as an extra meteorological factor (4 seasons: winter, spring, summer, and autumn). For the children, we investigated the inclusion of having hay fever symptoms (yes/no) at the day of the examination as well as season.

For seniors and as examined in prior studies (38), we evaluated potential effect modification by inflammation markers (i.e., C-reactive protein as a continuous variable) and for blood pressure (hypertensive vs. not hypertensive). We conducted effect modification analyses through addition of a multiplicative interaction term between the exposure and the covariate of interest into the main model.

The models were estimated using restricted maximum likelihood (REML) and “nlminb” optimizer [R-package “state” (39)]. We employed the R functions varIdent() to define the variance function structure and lme() to fit the models [R-package “nlme” (35, 40)]. All statistical analyses were conducted in R version 4.3 (R Core Team) (39).

## 3. Results

In total, 47 seniors (37 females, mean age = 76.7) and 47 children (27 females, mean age = 8.1) were included in the analyses (Table 1). Six seniors were obese and a third were former smokers. The majority were on heart medication (61.7%) and half had hypertension. Among children, only one was obese, none reported asthma problems, and none were on regular medication.

**Table 1:** Characteristics of the study population at the initial examination.

Characteristics	Seniors mean (SD) or *n* (%)	Children mean (SD) or *n* (%)
Number of subjects	47	47
Sex (females)	37 (78.7)	27 (57.4)
Age (year)	76.7 (9.6)	8.1 (1.2)
Height (cm)	164 (6.85)	133 (9.4)
Weight (kg)	69.1 (12.3)	28.5 (5.7)
BMI (kg/m^2^)	25.6 (4.1)	16.1 (1.9)
Obesity status		
Underweight	1 (2.1)	4 (8.5)
Normal	24 (51.1)	37 (78.7)
overweight	16 (34.0)	5 (10.6)
Obese	6 (12.8)	1 (2.1)
Former smokers	15 (31.9)	–
On heart medication	29 (61.7)	–
Hypertension (yes)	23 (48.9)	0

Seniors were followed up between May 2000 and June 2001 with a total of 899 available observations on HRV and an average of 19.1 observations per senior. Of the 899 observations, 603 and 578 had complete UFP and fine PM measures, respectively, with no missing values (Supplementary Figure S1A). The mean values for SDNN and RMSSD were 30.7 ms (SD = 14.3) and 22.7 ms (SD = 12.2) respectively (Table 2). For the seniors, the mean UFP exposure was 27,471 (SD = 15,100) n/ml. Very high positive correlations were found between PM_2.5_ and PM_10_ with medium to high positive correlations between PM_2.5_, PM_10_, NO_2_ and UFP and medium to high negative correlations between UFP and O_3_ (Table 3).

**Table 2:** HRV outcome characteristics averaged over examination days.

HRV characteristics	Seniors		Children	
	Mean (SD)	Median (IQR)	Mean (SD)	Median (IQR)
SDNN (ms)	30.7 (14.3)	28.4 (17.9)	49.3 (16.3)	46.7 (20.9)
RMSSD (ms)	22.7 (12.2)	19.4 (16.6)	46.3 (18.8)	42.8 (25.6)
LF power (ms^2^)	274 (338)	151 (257)	807 (560)	657 (630)
HF power (ms^2^)	144 (190)	77.8 (144)	664 (452)	588 (624)
LF/HF (%, higher is worse)	2.5 (1.9)	2.1 (2.2)	1.6 (1.3)	1.3 (1.2)

SDNN, standard deviation of normal to normal intervals; RMSSD, root mean square of successive differences between normal heartbeats; LF, low frequency power; HF, high frequency power; LF/HF, ratio of low to high frequency power and HRV, heart rate variability.

**Table 3:** Air pollution characteristics averaged over examination days.

	Descriptive statistics							Spearman correlation coefficients			
	Mean (SD)	Min	P25	P50	P75	Max	IQR	PM_2.5_	PM_10_	NO_2_	O_3_
Seniors											
UFP (n/ml)	27,471.4 (15,099.8)	5,981.3	16,609.4	23,271.8	32,345.1	96,343.7	15,735.7	0.610	0.563	0.698	−0.600
PM_2.5_ (µg/m^3^)	14.9 (8.9)	1.8	9.4	13.0	18.1	63.0	8.7		0.937	0.506	−0.438
PM_10_ (µg/m^3^)	19.9 (10.7)	4.0	13.0	18.5	23.6	71.4	10.6			0.500	−0.391
NO_2_ (µg/m^3^)	28.3 (12.1)	7.4	19.3	24.8	36.3	70.5	17.0				−0.792
O_3_ (µg/m^3^)	54.5 (31.8)	3.6	27.8	55.3	81.8	148.9	54.0				
Temperature (°C)	12.1 (7.7)	−6.7	5.4	12.1	18.5	28.2	13.1				
Water vapor pressure (hPa)	10.5 (3.5)	4.9	7.5	10.3	13.3	20.1	5.8				
Precipitation (mm)	1.1 (3)	0.0	0.0	0.0	0.8	35.7	0.8				
Children											
UFP (n/ml)	19,872.4 (5,481.7)	8,857.8	16,241.3	19,582.1	22,698.6	34,881.9	6,457.3	−0.087	−0.005	0.002	0.316
PM_2.5_ (µg/m^3^)	16.4 (13.3)	0.4	7.8	12.0	20.9	94.2	13.1		0.950	0.363	−0.146
PM_10_ (µg/m^3^)	21.5 (15.2)	1.8	11.2	17.0	27.5	102.9	16.3			0.435	−0.130
NO_2_ (µg/m^3^)	27.8 (16)	6.5	17.4	23.9	35.4	148.3	18.0				−0.644
O_3_ (µg/m^3^)	43.2 (27.9)	3.0	23.1	38.9	56.0	142.9	32.9				
Temperature (°C)	10.1 (8.6)	−11.3	4.2	10.3	16.5	28.6	12.3				
Water vapor pressure (hPa)	10.2 (4.1)	4.0	6.8	9.2	12.7	22.7	5.9				
Precipitation (mm)	1.1 (4.1)	0.0	0.0	0.0	0.2	58.9	0.2				

UFP, ultrafine particles; PM, particulate matter.

Children were followed up between September 2001 and July 2002 with a total of 925 available observations on HRV and an average of 19.7 observations per child. Of the 925 observations, only 325 had complete UFP measures but 710 had complete fine PM measures (Supplementary Figure S1B). The mean values for SDNN and RMSSD were 49.3 ms (SD = 16.3) and 46.3 ms (SD = 18.8) respectively (Table 2). The mean UFP exposure was 19,872 (SD = 5,482) n/ml. Similar to the seniors, a very high positive correlation was observed between PM_2.5_ and PM_10_. However very low negative correlations were present between PM_2.5_, PM_10_, NO_2_ and UFP (Table 3).

### 3.1. Seniors

**Figure 1:**
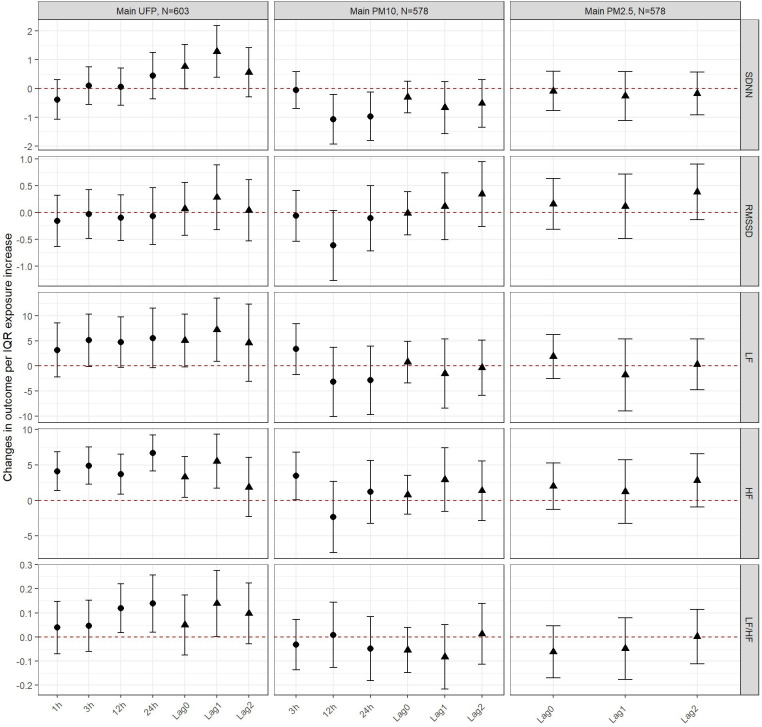
Changes in outcomes per IQR exposure increase to UFP, PM10 and PM2.5 among seniors. The circular estimates represent the hours (1, 3, 12 or 24 hours) after exposure and the triangular estimates represent the daily lags (0, 1, 2).

In UFP single pollutant models among seniors (Figure 1 and Supplementary Table S3), we observed an increase in SDNN at lags 0 and 1 day. In contrast, at short term exposures to PM_10_, we observed decreases in SDNN (lags 12 and 24 h, and lag 1 day). No associations were found between UFP exposure and RMSSD. PM_10_ (12 h lag) was associated with a clear decrease in RMSSD. In relation to the frequency-domain outcomes, UFP exposure was associated with consistent increases in both LF and HF powers at all lags, whereas for PM_10_, only very acute exposure was associated with a transient increase in LF and HF. UFP exposures (lags 12 and 24 h and lags 1 and 2 days) were positively associated with the LF/HF ratio, but not PM_10_ was not. No associations were observed between exposure to PM_2.5_ and any outcomes.

**Figure 2:**
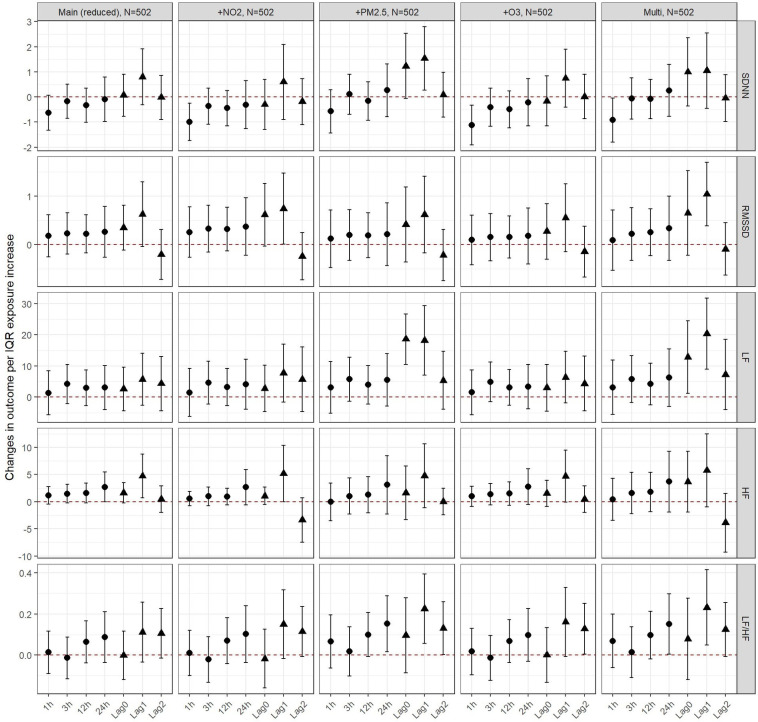
Changes in outcomes per IQR exposure increase to UFP, PM10 and PM2.5 among seniors - two and multi-pollutant models. The circular estimates represent the hours (1, 3, 12 or 24 hours) after exposure and the triangular estimates represent the daily lags (0, 1, 2).

In UFP models, we observed robust results after adjusting for NO_2_ and O_3_ throughout the 5 different outcomes. Results were also robust to adjustment for PM_2.5_ with the exception of a more pronounced increase in SDNN and LF at lag 0 and lag 1, thus also affecting the estimates at lag 0 and 1 in multipollutant modelling (Figure 2 and Supplementary Table S4). In PM_10_ models, results were robust to adjustment for NO_2_ and O_3_ in two and multipollutant modelling (Supplementary Figure S3).

### 3.2. Children

**Figure 3:**
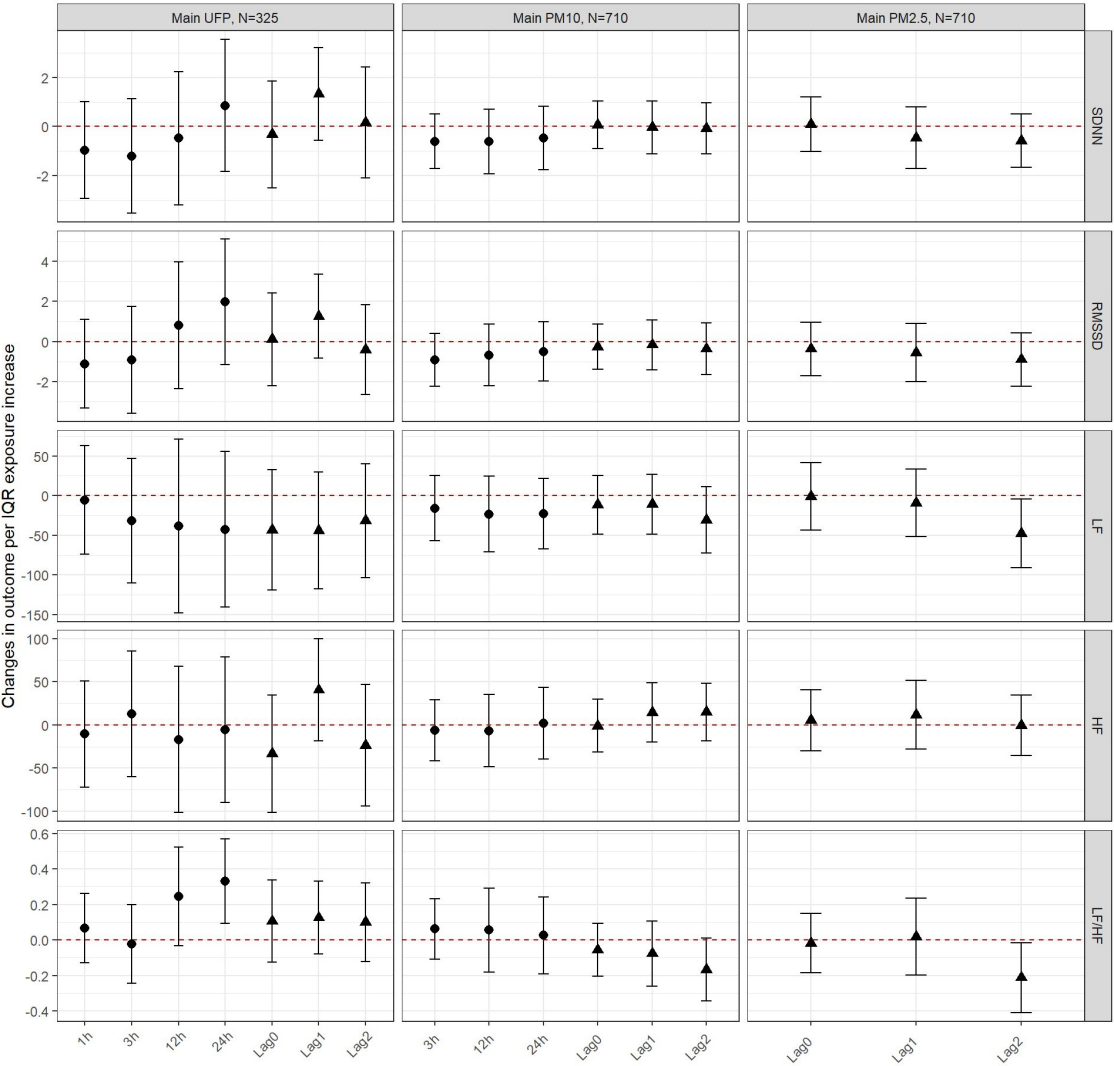
Changes in outcomes per IQR exposure increase to UFP, PM10 and PM2.5 among children. The circular estimates represent the hours (1, 3, 12 or 24 hours) after exposure and the triangular estimates represent the daily lags (0, 1, 2).

Among children no clear associations were found between UFP and fine PM and the time-domain outcomes (Figure 3 and Supplementary Table S5). While point estimates for UFP were consistently below 0 for LF, all CIs were large and overlapped the null. In contrast, no patterns could be observed for UFP and HF. Similarly for PM_10_, consistent but weak decreases were seen for LF but no pattern for HF. Short-term exposures to UFP (12 and 24 h) were positively associated with the LF/HF ratio. PM_2.5_ and PM_10_ were both negatively associated with the ratio at lag 2.

**Figure 4:**
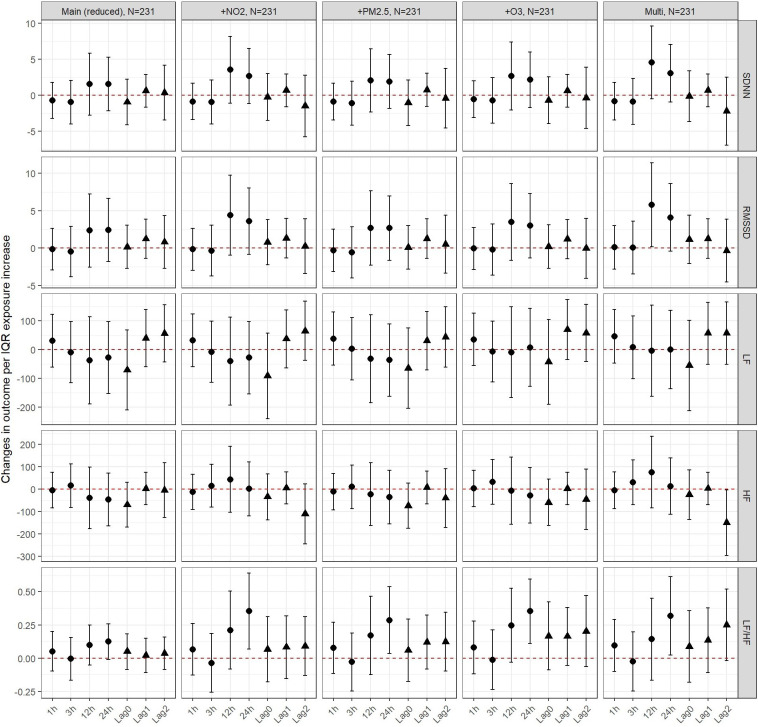
Changes in outcomes per IQR exposure increase to UFP, PM10 and PM2.5 among children - two and multi-pollutant models. The circular estimates represent the hours (1, 3, 12 or 24 hours) after exposure and the triangular estimates represent the daily lags (0, 1, 2).

In UFP models, results were robust to adjustment for PM_2.5_ and O_3_ throughout the 5 different outcomes. Models were also robust to adjustment for NO_2_ with the exception of a more pronounced increase in SDNN and LF/HF ratio at lag 12 and 24 h thus also affecting the estimates in multipollutant modelling (Figure 4 and Supplementary Table S6). In PM_10_ models, results were robust to adjustment for NO_2_ and O_3_ in two and multipollutant modelling (Supplementary Figure S4).

### 3.3. Sensitivity and effect modification analyses

Sensitivity analyses showed that UFP and PM_10_ results were robust to adjustment for heart medication and season among seniors (Supplementary Figures S5, S6). Among children, model results for UFP and PM_10_ were largely unchanged after adjusting for season and hay fever (Supplementary Figures S7, S8). There were no clear differences in the effects when it came to analyses by hypertension (Supplementary Figures S9, S10) or CRP (results not shown) among seniors in any of the exposures.

## 4. Discussion

### 4.1. Our findings

Among seniors, we observed positive associations between SDNN, LF, HF and LF/HF ratio and short-term exposure to UFP (hourly and daily lags) in contrast to decreases in SDNN and RMSSD after exposure to PM_10_. Generally, the association between fine PM and all 5 HRV outcomes was less clear. For two and multipollutant modelling, we generally found UFP associations were robust to adjustment. Among children, we observed inconsistent associations between UFP and all HRV outcomes and mostly null associations between PM_2.5_ and PM_10_ and HRV outcomes. Results were robust for multipollutant modelling in general, however, adjustment for NO_2_ enhanced some patterns for the association of UFP with SDNN and LF/HF (UFP lag 12 and 24 h).

### 4.2. Comparing with the literature

In terms of short-term exposure to UFP, and in contrast to our findings, a recent meta-analysis by Zhang et al. (22) showed decreases in SDNN, RMSSD, LF and LF/HF within 6 h after exposure. Moreover, they found that daily lags on the same day or preceding days were not associated with HRV which is in contrast with our results of a positive association with SDNN, LF, HF and LF/HF. In terms of short-term exposure to fine PM, previous studies on the general population show inconsistent associations with HRV outcomes. Some observed decreases in time-domains (21, 41) and frequency-domain outcomes (20, 27, 42, 43), but also others observed increases in both domains (43–45) or no associations at all (46–48) as seen in our study.

Further, a very limited number of studies have looked at the exposure of ambient short-term UFP and fine PM on HRV outcomes among children. A study in California demonstrated that adolescents residing in neighborhoods characterized by higher concentrations of PM_2.5_ had a decrease in HRV specifically in HF band in response to social stress (49). Saenen et al. (30), examined the association between fine PM exposure at home and school on SDNN, RMSSD, HF and LF among school children aged between 9 and 12 years old and found a decrease in LF with an increase in outdoor PM_10_ exposure as well as decreases in RMSSD, HF and LF with an increase in indoor PM_10_ exposure. In contrast, we observed no consistent and clear associations between fine PM exposure and HRV. Chen et al. (29) investigated the short-term effects of indoor UFP on children aged between 11 and 14 years old in schools in Beijing and found decreases in SDNN at lag 1 and decreases in SDNN, LF and HF at lag 2. Similarly, we found decreases in LF at most hourly and daily lags.

### 4.3. Multipollutant modelling and sensitivity analyses

Our results for two and multipollutant models showed mostly robust results. A review on ambient air pollution and HRV conducted by Buteau and Goldberg (50) observed some decreases in HRV after the exposure to NO_2_ ranging between 5 min to 5 days prior measurements. In our study and among seniors, the correlations between pollutants were high. Even though UFP was correlated with the co-pollutants, including NO_2_, the robustness in our results indicates that there was no major collinearity influencing the reliability of the two and multi-pollutant models and that the associations seen between UFP and HRV were independent of fine PM, NO_2_ and O_3_. Moreover, among children, we observed a weaker correlation between UFP and the co-pollutants compared to the seniors. We adjusted our analyses for NO_2_ as surrogate for traffic related air pollution (TRAP), thus reducing confounding of the air pollutant and HRV associations by sources of traffic. However, the potential for residual confounding by traffic remains due to the imperfect correlation between NO_2_ and TRAP.. In terms of adjusting for O_3_ and as observed by a recent systematic review, it is important to consider adjustment for O_3_ since an association has been observed between short-term exposure to O_3_ and a decrease in HRV (51). Moreover, in a study conducted on HRV in a two-pollutant model including PM_2.5_ and O_3_, it was concluded that PM_2.5_ influenced HRV rather than O_3_ (31). We obtained comparable conclusions where, after the adjustment of O_3_ in two and multipollutant modelling, the results were robust leading to the affirmation of the effects of UFP and fine PM.

It is important to note that half of the seniors were on heart medication and the previous literature observed a decrease in HRV activity among people with heart disease (31, 52). This might explain the low SDNN in comparison to other studies in the general population. Upon adjusting for heart medication, the estimates were robust.

### 4.4. Risk of exposure misclassification

It is important to note that the placement of the pollutant measurement stations may lead to pollutant-specific exposure misclassification. In particular, among seniors, the fine PM measurement sampler was located in the park of the care home whereas for UFP, the counter was placed 3 km. away from the care home. For NO2 and O3, the measuring station was located 9 km north of the care home. We therefore expect a higher degree of exposure misclassification for UFP and NO2. However, simultaneous measurements of UFP at the care home showed that the temporal concentration changes at both locations were very similar. Among children, the exposure measurement does not account for exposures of children at home but only at school, potentially contributing to the inconsistent results in children.

### 4.5. Biological mechanisms

Previous studies have demonstrated that exposure to UFP and fine PM causes both inflammation and oxidative stress that can contribute to cardiovascular disease and eventually cardiovascular mortality (53). A number of experiments have shown the biological mechanisms behind the association of UFP and fine PM with cardiovascular diseases at the molecular level. The very first contact of the particles is with the nasal and pulmonary receptors in the respiratory epithelium that will lead to a disturbance of the autonomic dysfunction and perturbations in the heart rate. Secondly, oxidative stress initiates an inflammatory response by the release of biologically active intermediates. This localized imbalance might also lead to an oxidative stress response and eventually lung inflammation. HRV is an indicator of the ANS that depends on the SNS and PNS for transferring information and where both systems interplay to regulate HRV (53). An imbalance in the function of the SNS and PNS could have opposite effects on HRV leading either to an increase or a decrease in HRV. More specifically, the effects of an exposure to UFP and fine PM could be mediated either through the SNS leading to decreases in HRV or through the PNS leading to increases in HRV (33). The SDNN and LF band reflect both SNS and PNS activities while the RMSSD and the HF band reflect PNS activity. We therefore consider the LF/HF as a ratio between SNS/PNS.

Among seniors we observed a general decrease in SDNN, RMSSD and HF after the exposure to PM_10_ which would suggest a sympathetic response. In line with this, we also observed increases in the LF/HF ratio upon the exposure to UFP. This suggests greater SNS than PNS responses, which coincides with previous studies where greater SNS responses played an important role in sudden heart attacks or arrhythmia (22). The same response was observed among children with increase in the LF/HF ratio upon the exposure to UFP leading to the risks of arrythmia and heart disease (22).

### 4.6. Strengths and limitations

This study has several strengths, firstly the ability to consider a range of exposure windows for all air pollutants and thus the assessment of temporal exposure patterns, both hourly and daily lags. Second the availability of data on co-pollutants allowed us to adjust for those in two and multipollutant modelling. Thirdly, the design of Corpuscula allowed for repeated-measures. While the number of participants was small, we were able to analyze a large number of observations per participant, which was enough to detect some associations among the seniors.

One main limitation is the small number of observations we had among children in terms of UFP measures and when adjusting for multi-pollutants. Because of the large number of missing values, the weighted models did not converge, and we had to report unweighted results, thus the confidence intervals were very wide and we were not able to detect clear associations. Another limitation was that we did not have personal exposure measures for NO_2_ and O_3_, since these co-pollutants were not measured at the school or the care-home but instead 7–8 km away from the actual research area. Thus, our ability to detect accurate associations for these pollutants in multipollutant modelling might have been reduced. A further limitation is that the study was carried among seniors and children, and the results may not be transferable to other age groups or to the general population. Nevertheless, these are populations traditionally thought to be particularly vulnerable to the effects of air pollution.

## 5. Conclusions

Overall, among seniors, we observed associations of UFP and PM_10_ exposure with sympathetic responses of the ANS, which play an important role in sudden heart attacks or arrhythmia. Among children we found more inconsistent associations between UFP and a delayed increase in HRV. Adjusting for co-pollutants including NO_2_ and O_3_ yielded robust results.

## Data Availability

The raw data supporting the conclusions of this article will be made available by the authors, without undue reservation.
