# Supplementary material for: Short-term exposure to ultrafine and fine particulate matter with multipollutant modelling on heart rate variability among seniors and children from the CorPuScula (coronary, pulmonary, sanguis) longitudinal study in Germany
Source: Front Epidemiol. 2023 Nov 8;3:1278506. doi: 10.3389/fepid.2023.1278506 (PMC10910943; doi:10.3389/fepid.2023.1278506)
Supplement: Supplementary file 1 [file Datasheet1.pdf]

Supplementary Figure 1: Flow diagram describing the original samples versus the ones used in the analyses (A: Seniors and B: Children)

A: Seniors

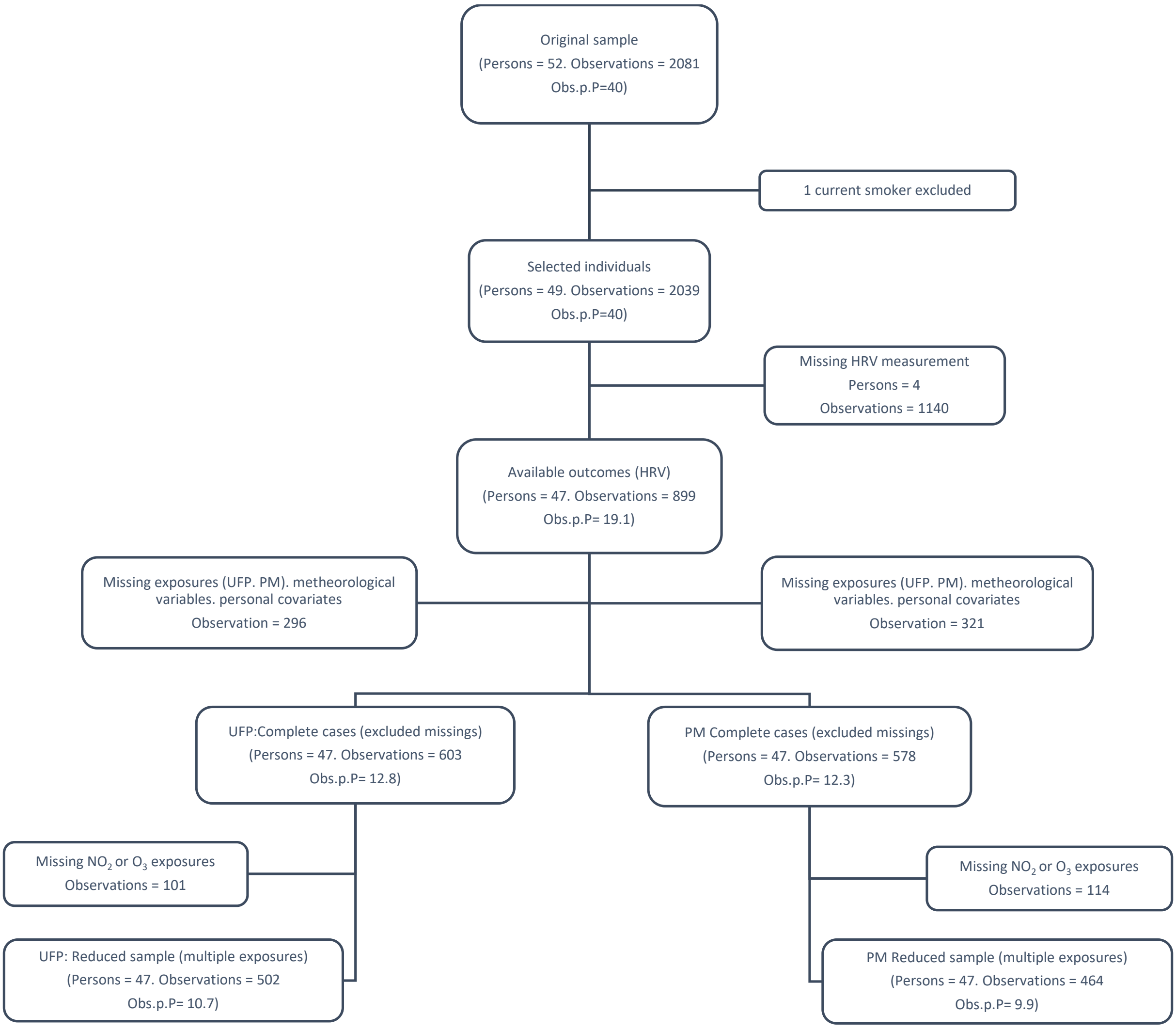

B: Children

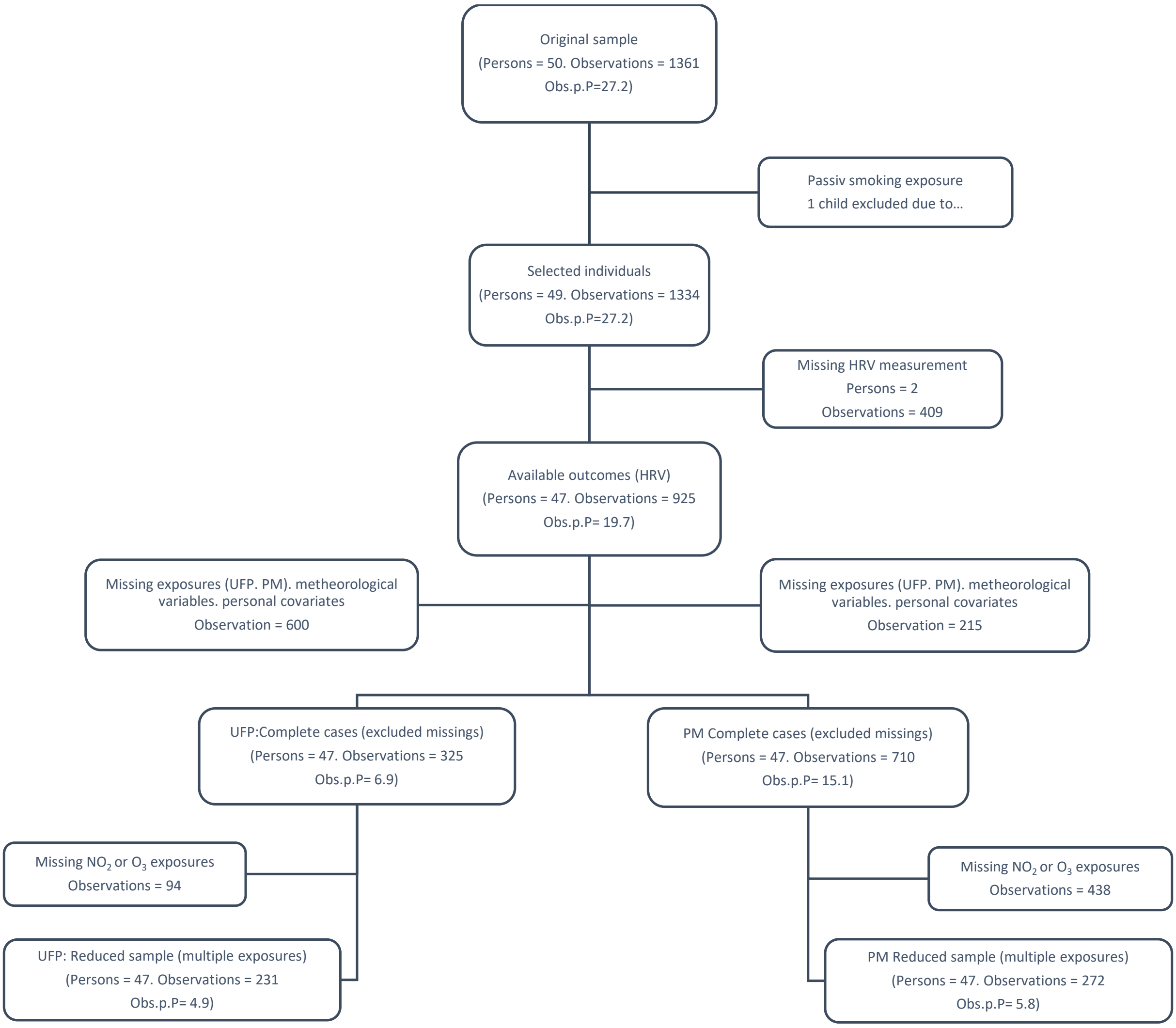
Original sample
(Persons = 50. Observations = 1361
Obs.p.P=27.2)
Passiv smoking exposure
1 child excluded due to…
Selected individuals
(Persons = 49. Observations = 1334
Obs.p.P=27.2)
Missing HRV measurement
Persons = 2
Observations = 409
Available outcomes (HRV)
(Persons = 47. Observations = 925
Obs.p.P= 19.7)
Missing exposures (UFP. PM). metheorological variables. personal covariates
Observation = 600
Missing exposures (UFP. PM). metheorological variables. personal covariates
Observation = 215
UFP:Complete cases (excluded missings)
(Persons = 47. Observations = 325
Obs.p.P= 6.9)
PM Complete cases (excluded missings)
(Persons = 47. Observations = 710
Obs.p.P= 15.1)
Missing NO_2 or O_3 exposures
Observations = 94
Missing NO_2 or O_3 exposures
Observations = 438
UFP: Reduced sample (multiple exposures)
(Persons = 47. Observations = 231
Obs.p.P= 4.9)
PM Reduced sample (multiple exposures)
(Persons = 47. Observations = 272
Obs.p.P= 5.8)

Supplementary Figure 2: DAG for the association of PM/UFP and HRV outcomes (A: Seniors; B: Children)

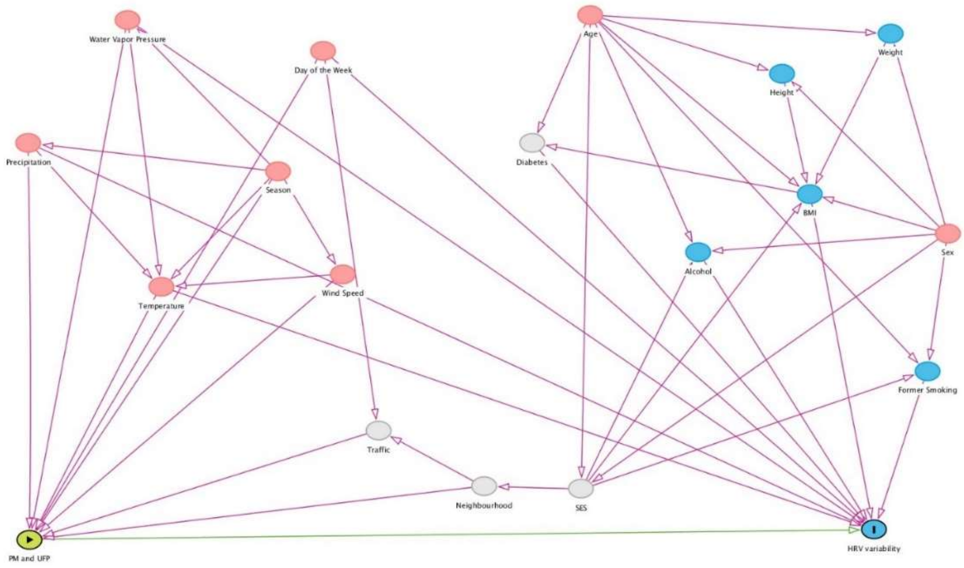

A: Minimal sufficient adjustment set for estimating the total effect of PM and UFP on HRV variability among the senior group: age, alcohol, BMI, Day of the Week, Precipitation, Temperature, Water Vapor Pressure (Abbreviations: PM, particulate matter; UFP, ultrafine particles; HRV, heart rate variability; SES, socioeconomic status; BMI, body mass index)

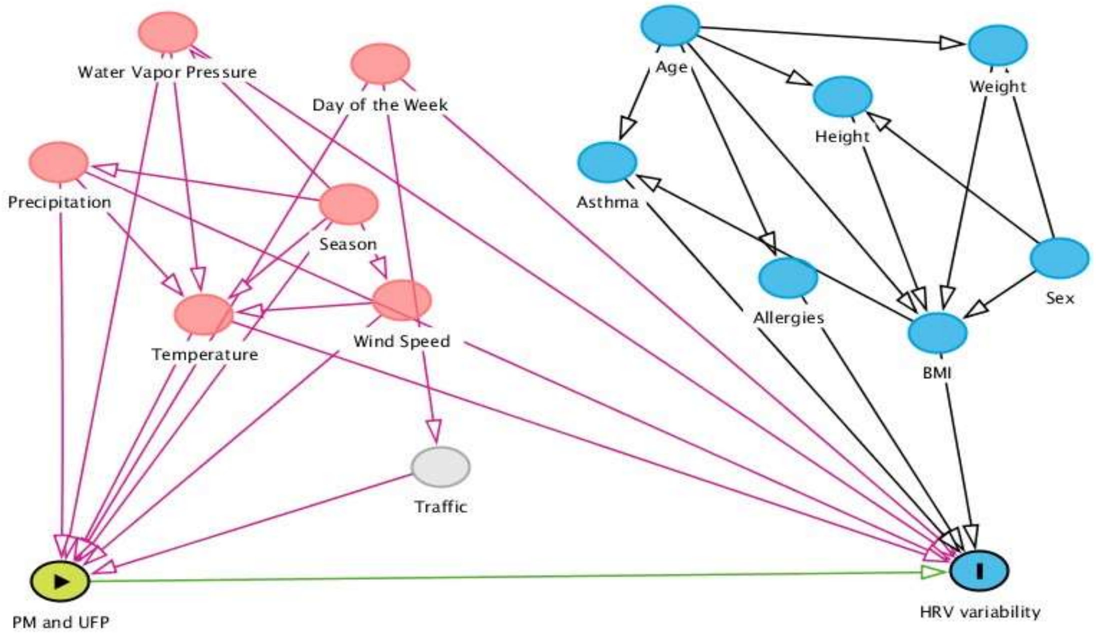

B: Minimal sufficient adjustment set for estimating the total effect of PM and UFP on HRV variability among the children group: day of the week, precipitation, temperature, water vapor pressure (Abbreviations: PM, particulate matter; UFP, ultrafine particles; HRV, heart rate variability; BMI, body mass index)

Supplementary Table 1: Correlation Matrix between the Outcomes among Seniors and Children Separately

Seniors	SDNN	RMSSD	LF	HF	LF/HF
SDNN	1	0.7	0.773	0.624	0.153
RMSSD	0.7	1	0.548	0.789	-0.261
LF	0.773	0.548	1	0.599	0.287
HF	0.624	0.789	0.599	1	-0.248
LF/HF	0.153	-0.261	0.287	-0.248	1
**Children**	**SDNN**	**RMSSD**	**LF**	**HF**	**LF/HF**
SDNN	1	0.861	0.836	0.727	-0.109
RMSSD	0.861	1	0.652	0.739	-0.288
LF	0.836	0.652	1	0.544	0.17
HF	0.727	0.739	0.544	1	-0.446
LF/HF	-0.109	-0.288	0.17	-0.446	1

Abbreviations; DNN, standard deviation of normal to normal intervals; RMSSD, root mean square of successive differences between normal heartbeats; LF, low frequency power; HF, high frequency power; LF/HF, ratio of Low to high frequency power

Supplementary Table 2: The Lags in Days for Meteorological Parameters Used in the Adjusted Models

Outcome	**Vapor Pressure (spline=0)**		**Precipitation (spline=6)**		**Temperature (spline=6)**	
	**Seniors**	**Children**	**Seniors**	**Children**	**Seniors**	**Children**
HF	0 day	0 day	1 day	2 days	0 day	0 day
LF	1 day	2 days	0 day	2 days	1 day	0 day
LF/HF	0 day	0 day	2 days	0 day	1 day	1 day
RMSSD	0 day	0 day	1 day	0 day	0 day	0 day
SDNN	0 day	0 day	2 days	0 day	0 day	0 day

Abbreviations: HF, high frequency power; LF, low frequency power; LF/HF, ratio of Low to high frequency power; RMSSD, root mean square of successive differences between normal heartbeats; SDNN, standard deviation of normal to normal intervals

Supplementary Table 3: The Lags in Days for NO_2, O_3 and $PM_{2.5}$ Used in the Multi-Pollutant Models

	**NO_2**		**O_3**		**$PM_{2.5}$**	
	**Seniors**	**Children**	**Seniors**	**Children**	**Seniors**	**Children**
HF	2 days	0 day	1 day	2 days	0 day	0 day
LF	2 days	0 day	0 day	1 day	0 day	1 day
LF/HF	0 day	1 day	1 day	2 days	0 day	2 days
RMSSD	1 day	0 day	0 day	2 days	0 day	0 day
SDNN	0 day	0 day	0 day	2 days	0 day	0 day

Abbreviations: HF, high frequency power; LF, low frequency power; LF/HF, ratio of Low to high frequency power; RMSSD, root mean square of successive differences between normal heartbeats; SDNN, standard deviation of normal to normal intervals

Supplementary Table 4: Change in Outcomes per IQR exposure increase to UFP, PM_{10} and $PM_{2.5}$ among Seniors: estimates and 95% CI for both the crude and the main models

**Outcome**	**Exposure**	**Model**	**Estimate**	**CI low**	**CI high**
SDNN	UFP 1h	Crude	-0.493	-1.059	0.074
SDNN	UFP 1h	Main	-0.383	-1.065	0.298
SDNN	UFP 3h	Crude	-0.185	-0.725	0.355
SDNN	UFP 3h	Main	0.094	-0.555	0.744
SDNN	UFP 12h	Crude	-0.237	-0.758	0.284
SDNN	UFP 12h	Main	0.059	-0.589	0.707
SDNN	UFP 24h	Crude	0.094	-0.537	0.726
SDNN	UFP 24h	Main	0.447	-0.355	1.249
SDNN	UFP lag0	Crude	0.653	0.056	1.250
SDNN	UFP lag0	Main	0.763	-0.009	1.534
SDNN	UFP lag1	Crude	1.136	0.438	1.835
SDNN	UFP lag1	Main	1.286	0.390	2.181
SDNN	UFP lag2	Crude	0.359	-0.318	1.036
SDNN	UFP lag2	Main	0.557	-0.297	1.410
SDNN	PM_{10} 3h	Crude	-0.088	-0.678	0.501
SDNN	PM_{10} 3h	Main	-0.059	-0.696	0.578
SDNN	PM_{10} 12h	Crude	-0.654	-1.371	0.064
SDNN	PM_{10} 12h	Main	-1.072	-1.932	-0.212
SDNN	PM_{10} 24h	Crude	-0.432	-1.134	0.270
SDNN	PM_{10} 24h	Main	-0.967	-1.807	-0.128
SDNN	PM_{10} lag0	Crude	-0.135	-0.629	0.360
SDNN	PM_{10} lag0	Main	-0.296	-0.847	0.255
SDNN	PM_{10} lag1	Crude	-0.072	-0.833	0.689
SDNN	PM_{10} lag1	Main	-0.666	-1.570	0.237
SDNN	PM_{10} lag2	Crude	-0.290	-0.993	0.413
SDNN	PM_{10} lag2	Main	-0.517	-1.343	0.308
SDNN	$PM_{2.5}$ lag0	Crude	0.223	-0.341	0.787
SDNN	$PM_{2.5}$ lag0	Main	-0.087	-0.764	0.590
SDNN	$PM_{2.5}$ lag1	Crude	0.236	-0.521	0.993
SDNN	$PM_{2.5}$ lag1	Main	-0.261	-1.106	0.585
SDNN	$PM_{2.5}$ lag2	Crude	0.077	-0.612	0.766
SDNN	$PM_{2.5}$ lag2	Main	-0.173	-0.913	0.567
RMSSD	UFP 1h	Crude	-0.006	-0.377	0.365
RMSSD	UFP 1h	Main	-0.161	-0.640	0.319
RMSSD	UFP 3h	Crude	0.067	-0.284	0.419
RMSSD	UFP 3h	Main	-0.030	-0.483	0.423
RMSSD	UFP 12h	Crude	-0.169	-0.509	0.171
RMSSD	UFP 12h	Main	-0.099	-0.525	0.328
RMSSD	UFP 24h	Crude	-0.172	-0.588	0.245
RMSSD	UFP 24h	Main	-0.067	-0.597	0.462
RMSSD	UFP lag0	Crude	0.051	-0.341	0.442
RMSSD	UFP lag0	Main	0.069	-0.424	0.562
RMSSD	UFP lag1	Crude	0.089	-0.383	0.560
RMSSD	UFP lag1	Main	0.283	-0.323	0.888
RMSSD	UFP lag2	Crude	0.253	-0.179	0.684

RMSSD	UFP lag2	Main	0.039	-0.534	0.613
RMSSD	PM_{10} 3h	Crude	-0.230	-0.655	0.196
RMSSD	PM_{10} 3h	Main	-0.061	-0.536	0.415
RMSSD	PM_{10} 12h	Crude	-0.589	-1.093	-0.085
RMSSD	PM_{10} 12h	Main	-0.617	-1.269	0.034
RMSSD	PM_{10} 24h	Crude	-0.311	-0.793	0.172
RMSSD	PM_{10} 24h	Main	-0.108	-0.720	0.503
RMSSD	PM_{10} lag0	Crude	-0.173	-0.526	0.181
RMSSD	PM_{10} lag0	Main	-0.014	-0.418	0.391
RMSSD	PM_{10} lag1	Crude	-0.091	-0.606	0.424
RMSSD	PM_{10} lag1	Main	0.117	-0.506	0.740
RMSSD	PM_{10} lag2	Crude	0.217	-0.245	0.679
RMSSD	PM_{10} lag2	Main	0.344	-0.260	0.947
RMSSD	$PM_{2.5}$ lag0	Crude	-0.060	-0.455	0.335
RMSSD	$PM_{2.5}$ lag0	Main	0.157	-0.317	0.632
RMSSD	$PM_{2.5}$ lag1	Crude	0.066	-0.444	0.577
RMSSD	$PM_{2.5}$ lag1	Main	0.116	-0.487	0.720
RMSSD	$PM_{2.5}$ lag2	Crude	0.301	-0.137	0.740
RMSSD	$PM_{2.5}$ lag2	Main	0.382	-0.139	0.903
LF	UFP 1h	Crude	3.217	-0.546	6.981
LF	UFP 1h	Main	3.156	-2.238	8.549
LF	UFP 3h	Crude	4.000	0.521	7.478
LF	UFP 3h	Main	5.120	-0.112	10.353
LF	UFP 12h	Crude	2.762	-0.678	6.202
LF	UFP 12h	Main	4.746	-0.285	9.777
LF	UFP 24h	Crude	4.291	-0.043	8.625
LF	UFP 24h	Main	5.568	-0.345	11.480
LF	UFP lag0	Crude	4.382	0.727	8.038
LF	UFP lag0	Main	5.063	-0.216	10.342
LF	UFP lag1	Crude	5.535	0.995	10.076
LF	UFP lag1	Main	7.201	0.868	13.534
LF	UFP lag2	Crude	5.174	0.612	9.735
LF	UFP lag2	Main	4.633	-3.064	12.330
LF	PM_{10} 3h	Crude	1.268	-2.699	5.235
LF	PM_{10} 3h	Main	3.360	-1.699	8.419
LF	PM_{10} 12h	Crude	-2.477	-7.370	2.416
LF	PM_{10} 12h	Main	-3.209	-10.112	3.693
LF	PM_{10} 24h	Crude	-2.015	-6.258	2.228
LF	PM_{10} 24h	Main	-2.879	-9.719	3.961
LF	PM_{10} lag0	Crude	0.186	-2.949	3.322
LF	PM_{10} lag0	Main	0.760	-3.411	4.932
LF	PM_{10} lag1	Crude	-1.396	-6.043	3.250
LF	PM_{10} lag1	Main	-1.553	-8.477	5.371
LF	PM_{10} lag2	Crude	-1.027	-4.675	2.621
LF	PM_{10} lag2	Main	-0.366	-5.848	5.116
LF	$PM_{2.5}$ lag0	Crude	0.359	-3.025	3.743
LF	$PM_{2.5}$ lag0	Main	1.864	-2.493	6.221
LF	$PM_{2.5}$ lag1	Crude	-1.802	-6.671	3.067

LF	$PM_{2.5}$ lag1	Main	-1.794	-8.995	5.408
LF	$PM_{2.5}$ lag2	Crude	-0.578	-4.025	2.868
LF	$PM_{2.5}$ lag2	Main	0.305	-4.796	5.407
HF	UFP 1h	Crude	2.019	-0.123	4.161
HF	UFP 1h	Main	4.106	1.389	6.822
HF	UFP 3h	Crude	2.570	0.513	4.628
HF	UFP 3h	Main	4.886	2.256	7.516
HF	UFP 12h	Crude	1.319	-1.047	3.685
HF	UFP 12h	Main	3.700	0.881	6.519
HF	UFP 24h	Crude	1.390	-1.555	4.335
HF	UFP 24h	Main	6.678	4.163	9.193
HF	UFP lag0	Crude	0.447	-2.094	2.988
HF	UFP lag0	Main	3.296	0.402	6.190
HF	UFP lag1	Crude	0.378	-2.918	3.674
HF	UFP lag1	Main	5.521	1.725	9.317
HF	UFP lag2	Crude	4.296	1.439	7.154
HF	UFP lag2	Main	1.873	-2.303	6.049
HF	PM_{10} 3h	Crude	1.435	-1.415	4.285
HF	PM_{10} 3h	Main	3.448	0.107	6.790
HF	PM_{10} 12h	Crude	-3.197	-6.716	0.321
HF	PM_{10} 12h	Main	-2.348	-7.372	2.677
HF	PM_{10} 24h	Crude	-2.442	-5.783	0.899
HF	PM_{10} 24h	Main	1.193	-3.243	5.628
HF	PM_{10} lag0	Crude	0.052	-2.226	2.329
HF	PM_{10} lag0	Main	0.788	-1.927	3.502
HF	PM_{10} lag1	Crude	-0.639	-4.246	2.967
HF	PM_{10} lag1	Main	2.943	-1.545	7.431
HF	PM_{10} lag2	Crude	2.411	-0.800	5.622
HF	PM_{10} lag2	Main	1.380	-2.820	5.579
HF	$PM_{2.5}$ lag0	Crude	0.788	-1.772	3.348
HF	$PM_{2.5}$ lag0	Main	1.997	-1.262	5.257
HF	$PM_{2.5}$ lag1	Crude	-0.259	-3.961	3.443
HF	$PM_{2.5}$ lag1	Main	1.241	-3.243	5.724
HF	$PM_{2.5}$ lag2	Crude	2.955	-0.111	6.020
HF	$PM_{2.5}$ lag2	Main	2.832	-0.915	6.579
LF/HF	UFP 1h	Crude	0.012	-0.081	0.105
LF/HF	UFP 1h	Main	0.039	-0.070	0.148
LF/HF	UFP 3h	Crude	-0.001	-0.092	0.090
LF/HF	UFP 3h	Main	0.046	-0.060	0.152
LF/HF	UFP 12h	Crude	0.013	-0.067	0.093
LF/HF	UFP 12h	Main	0.119	0.018	0.221
LF/HF	UFP 24h	Crude	0.022	-0.073	0.117
LF/HF	UFP 24h	Main	0.138	0.019	0.257
LF/HF	UFP lag0	Crude	0.026	-0.076	0.129
LF/HF	UFP lag0	Main	0.049	-0.075	0.174
LF/HF	UFP lag1	Crude	0.053	-0.062	0.167
LF/HF	UFP lag1	Main	0.139	0.002	0.276
LF/HF	UFP lag2	Crude	0.036	-0.058	0.130

LF/HF	UFP lag2	Main	0.097	-0.029	0.224
LF/HF	PM_{10} 3h	Crude	0.010	-0.082	0.101
LF/HF	PM_{10} 3h	Main	-0.032	-0.137	0.073
LF/HF	PM_{10} 12h	Crude	0.090	-0.022	0.203
LF/HF	PM_{10} 12h	Main	0.008	-0.127	0.143
LF/HF	PM_{10} 24h	Crude	0.065	-0.043	0.174
LF/HF	PM_{10} 24h	Main	-0.049	-0.182	0.085
LF/HF	PM_{10} lag0	Crude	0.016	-0.068	0.099
LF/HF	PM_{10} lag0	Main	-0.055	-0.149	0.039
LF/HF	PM_{10} lag1	Crude	0.040	-0.072	0.152
LF/HF	PM_{10} lag1	Main	-0.083	-0.216	0.050
LF/HF	PM_{10} lag2	Crude	0.063	-0.032	0.159
LF/HF	PM_{10} lag2	Main	0.013	-0.114	0.139
LF/HF	$PM_{2.5}$ lag0	Crude	0.003	-0.094	0.099
LF/HF	$PM_{2.5}$ lag0	Main	-0.062	-0.170	0.046
LF/HF	$PM_{2.5}$ lag1	Crude	0.057	-0.047	0.161
LF/HF	$PM_{2.5}$ lag1	Main	-0.049	-0.176	0.079
LF/HF	$PM_{2.5}$ lag2	Crude	0.035	-0.058	0.127
LF/HF	$PM_{2.5}$ lag2	Main	0.002	-0.112	0.115

Abbreviations: UFP, ultrafine particles; PM, particulate matter; SDNN, standard deviation of normal to normal intervals; RMSSD, root mean square of successive differences between normal heartbeats; LF, low frequency power; HF, high frequency power; LF/HF, ratio of Low to high frequency power

Supplementary Table 5: Change in Outcomes per IQR exposure increase to UFP among Seniors: estimates and 95% CI for two and multi-pollutant models

Outcome	Exposure	Model	Estimate	CI low	CI high
SDNN	UFP 1h	Main. reduced	-0.628	-1.321	0.064
SDNN	UFP 3h	Main. reduced	-0.167	-0.849	0.515
SDNN	UFP 12h	Main. reduced	-0.332	-1.012	0.348
SDNN	UFP 24h	Main. reduced	-0.092	-0.980	0.797
SDNN	UFP lag0	Main. reduced	0.068	-0.771	0.908
SDNN	UFP lag1	Main. reduced	0.799	-0.320	1.918
SDNN	UFP lag2	Main. reduced	-0.014	-0.890	0.862
SDNN	UFP 1h	Main+NO_2	-0.992	-1.732	-0.251
SDNN	UFP 3h	Main+NO_2	-0.366	-1.086	0.354
SDNN	UFP 12h	Main+NO_2	-0.445	-1.142	0.253
SDNN	UFP 24h	Main+NO_2	-0.308	-1.262	0.646
SDNN	UFP lag0	Main+NO_2	-0.298	-1.290	0.694
SDNN	UFP lag1	Main+NO_2	0.601	-0.892	2.093
SDNN	UFP lag2	Main+NO_2	-0.185	-1.096	0.726
SDNN	UFP 1h	Main + $PM_{2.5}$	-0.573	-1.431	0.285
SDNN	UFP 3h	Main + $PM_{2.5}$	0.106	-0.696	0.909
SDNN	UFP 12h	Main + $PM_{2.5}$	-0.161	-0.930	0.608
SDNN	UFP 24h	Main + $PM_{2.5}$	0.268	-0.781	1.317
SDNN	UFP lag0	Main + $PM_{2.5}$	1.229	-0.067	2.526
SDNN	UFP lag1	Main + $PM_{2.5}$	1.531	0.264	2.798
SDNN	UFP lag2	Main + $PM_{2.5}$	0.093	-0.804	0.989
SDNN	UFP 1h	Main+O_3	-1.119	-1.904	-0.334
SDNN	UFP 3h	Main+O_3	-0.408	-1.172	0.356
SDNN	UFP 12h	Main+O_3	-0.492	-1.224	0.241
SDNN	UFP 24h	Main+O_3	-0.211	-1.149	0.728
SDNN	UFP lag0	Main+O_3	-0.160	-1.156	0.835
SDNN	UFP lag1	Main+O_3	0.743	-0.408	1.893
SDNN	UFP lag2	Main+O_3	0.013	-0.869	0.896
SDNN	UFP 1h	Main+NO_2+O_3+$PM_{2.5}$	-0.916	-1.791	-0.041
SDNN	UFP 3h	Main+NO_2+O_3+$PM_{2.5}$	-0.056	-0.877	0.764
SDNN	UFP 12h	Main+NO_2+O_3+$PM_{2.5}$	-0.079	-0.861	0.703
SDNN	UFP 24h	Main+NO_2+O_3+$PM_{2.5}$	0.258	-0.776	1.292
SDNN	UFP lag0	Main+NO_2+O_3+$PM_{2.5}$	0.996	-0.355	2.348
SDNN	UFP lag1	Main+NO_2+O_3+$PM_{2.5}$	1.046	-0.449	2.540
SDNN	UFP lag2	Main+NO_2+O_3+$PM_{2.5}$	-0.048	-0.979	0.883
RMSSD	UFP 1h	Main. reduced	0.179	-0.258	0.617
RMSSD	UFP 3h	Main. reduced	0.225	-0.201	0.652
RMSSD	UFP 12h	Main. reduced	0.219	-0.176	0.614
RMSSD	UFP 24h	Main. reduced	0.261	-0.266	0.787
RMSSD	UFP lag0	Main. reduced	0.347	-0.119	0.813
RMSSD	UFP lag1	Main. reduced	0.624	-0.048	1.296
RMSSD	UFP lag2	Main. reduced	-0.209	-0.727	0.308
RMSSD	UFP 1h	Main+NO_2	0.255	-0.266	0.775
RMSSD	UFP 3h	Main+NO_2	0.325	-0.160	0.810

RMSSD	UFP 12h	Main+NO_2	0.319	-0.132	0.770
RMSSD	UFP 24h	Main+NO_2	0.370	-0.225	0.966
RMSSD	UFP lag0	Main+NO_2	0.617	-0.032	1.266
RMSSD	UFP lag1	Main+NO_2	0.742	0.009	1.474
RMSSD	UFP lag2	Main+NO_2	-0.244	-0.736	0.247
RMSSD	UFP 1h	Main + $PM_{2.5}$	0.116	-0.478	0.711
RMSSD	UFP 3h	Main + $PM_{2.5}$	0.191	-0.333	0.716
RMSSD	UFP 12h	Main + $PM_{2.5}$	0.189	-0.274	0.651
RMSSD	UFP 24h	Main + $PM_{2.5}$	0.213	-0.436	0.862
RMSSD	UFP lag0	Main + $PM_{2.5}$	0.409	-0.367	1.186
RMSSD	UFP lag1	Main + $PM_{2.5}$	0.616	-0.179	1.411
RMSSD	UFP lag2	Main + $PM_{2.5}$	-0.219	-0.749	0.312
RMSSD	UFP 1h	Main+O_3	0.092	-0.425	0.609
RMSSD	UFP 3h	Main+O_3	0.150	-0.336	0.637
RMSSD	UFP 12h	Main+O_3	0.154	-0.285	0.592
RMSSD	UFP 24h	Main+O_3	0.175	-0.403	0.752
RMSSD	UFP lag0	Main+O_3	0.268	-0.304	0.841
RMSSD	UFP lag1	Main+O_3	0.553	-0.149	1.254
RMSSD	UFP lag2	Main+O_3	-0.149	-0.674	0.376
RMSSD	UFP 1h	Main+NO_2+O_3+$PM_{2.5}$	0.086	-0.538	0.711
RMSSD	UFP 3h	Main+NO_2+O_3+$PM_{2.5}$	0.217	-0.329	0.763
RMSSD	UFP 12h	Main+NO_2+O_3+$PM_{2.5}$	0.253	-0.231	0.736
RMSSD	UFP 24h	Main+NO_2+O_3+$PM_{2.5}$	0.332	-0.332	0.997
RMSSD	UFP lag0	Main+NO_2+O_3+$PM_{2.5}$	0.648	-0.226	1.522
RMSSD	UFP lag1	Main+NO_2+O_3+$PM_{2.5}$	1.039	0.382	1.696
RMSSD	UFP lag2	Main+NO_2+O_3+$PM_{2.5}$	-0.096	-0.639	0.446
LF	UFP 1h	Main. reduced	1.382	-5.641	8.406
LF	UFP 3h	Main. reduced	4.204	-2.061	10.469
LF	UFP 12h	Main. reduced	3.033	-2.697	8.763
LF	UFP 24h	Main. reduced	3.100	-3.976	10.176
LF	UFP lag0	Main. reduced	2.591	-4.443	9.625
LF	UFP lag1	Main. reduced	5.710	-2.644	14.064
LF	UFP lag2	Main. reduced	4.352	-4.358	13.063
LF	UFP 1h	Main+NO_2	1.496	-6.199	9.191
LF	UFP 3h	Main+NO_2	4.632	-2.201	11.465
LF	UFP 12h	Main+NO_2	3.218	-2.744	9.181
LF	UFP 24h	Main+NO_2	4.140	-3.857	12.138
LF	UFP lag0	Main+NO_2	2.781	-4.623	10.186
LF	UFP lag1	Main+NO_2	7.736	-1.577	17.050
LF	UFP lag2	Main+NO_2	5.748	-4.647	16.144
LF	UFP 1h	Main + $PM_{2.5}$	3.106	-5.176	11.387
LF	UFP 3h	Main + $PM_{2.5}$	5.740	-1.342	12.822
LF	UFP 12h	Main + $PM_{2.5}$	3.945	-2.225	10.114
LF	UFP 24h	Main + $PM_{2.5}$	5.527	-2.882	13.936
LF	UFP lag0	Main + $PM_{2.5}$	18.635	10.549	26.721
LF	UFP lag1	Main + $PM_{2.5}$	18.185	7.063	29.308
LF	UFP lag2	Main + $PM_{2.5}$	5.359	-3.956	14.673
LF	UFP 1h	Main+O_3	1.517	-5.707	8.742

LF	UFP 3h	Main+O_3	4.908	-1.427	11.243
LF	UFP 12h	Main+O_3	3.101	-2.688	8.890
LF	UFP 24h	Main+O_3	3.378	-3.749	10.505
LF	UFP lag0	Main+O_3	2.978	-4.507	10.464
LF	UFP lag1	Main+O_3	6.370	-1.931	14.671
LF	UFP lag2	Main+O_3	4.333	-4.462	13.128
LF	UFP 1h	Main+NO_2+O_3+$PM_{2.5}$	3.137	-5.617	11.891
LF	UFP 3h	Main+NO_2+O_3+$PM_{2.5}$	5.786	-1.755	13.327
LF	UFP 12h	Main+NO_2+O_3+$PM_{2.5}$	4.236	-2.457	10.930
LF	UFP 24h	Main+NO_2+O_3+$PM_{2.5}$	6.237	-2.994	15.469
LF	UFP lag0	Main+NO_2+O_3+$PM_{2.5}$	12.855	1.226	24.483
LF	UFP lag1	Main+NO_2+O_3+$PM_{2.5}$	20.352	8.941	31.763
LF	UFP lag2	Main+NO_2+O_3+$PM_{2.5}$	7.236	-4.022	18.495
HF	UFP 1h	Main. reduced	1.155	-0.445	2.755
HF	UFP 3h	Main. reduced	1.452	-0.269	3.173
HF	UFP 12h	Main. reduced	1.605	-0.247	3.458
HF	UFP 24h	Main. reduced	2.720	-0.034	5.475
HF	UFP lag0	Main. reduced	1.638	-0.230	3.506
HF	UFP lag1	Main. reduced	4.726	0.716	8.737
HF	UFP lag2	Main. reduced	0.474	-1.994	2.942
HF	UFP 1h	Main+NO_2	0.583	-0.755	1.922
HF	UFP 3h	Main+NO_2	0.975	-0.714	2.664
HF	UFP 12h	Main+NO_2	0.930	-0.576	2.437
HF	UFP 24h	Main+NO_2	2.657	-0.604	5.918
HF	UFP lag0	Main+NO_2	1.062	-0.548	2.672
HF	UFP lag1	Main+NO_2	5.162	-0.031	10.355
HF	UFP lag2	Main+NO_2	-3.364	-7.453	0.725
HF	UFP 1h	Main + $PM_{2.5}$	-0.049	-3.525	3.427
HF	UFP 3h	Main + $PM_{2.5}$	1.033	-2.295	4.360
HF	UFP 12h	Main + $PM_{2.5}$	1.272	-2.075	4.619
HF	UFP 24h	Main + $PM_{2.5}$	3.121	-2.251	8.493
HF	UFP lag0	Main + $PM_{2.5}$	1.605	-3.336	6.546
HF	UFP lag1	Main + $PM_{2.5}$	4.764	-1.118	10.647
HF	UFP lag2	Main + $PM_{2.5}$	0.025	-2.454	2.504
HF	UFP 1h	Main+O_3	0.981	-0.906	2.868
HF	UFP 3h	Main+O_3	1.382	-0.599	3.362
HF	UFP 12h	Main+O_3	1.503	-0.653	3.660
HF	UFP 24h	Main+O_3	2.755	-0.548	6.058
HF	UFP lag0	Main+O_3	1.546	-0.856	3.948
HF	UFP lag1	Main+O_3	4.689	-0.097	9.476
HF	UFP lag2	Main+O_3	0.445	-1.994	2.883
HF	UFP 1h	Main+NO_2+O_3+$PM_{2.5}$	0.418	-3.451	4.287
HF	UFP 3h	Main+NO_2+O_3+$PM_{2.5}$	1.599	-2.198	5.396
HF	UFP 12h	Main+NO_2+O_3+$PM_{2.5}$	1.792	-1.837	5.422
HF	UFP 24h	Main+NO_2+O_3+$PM_{2.5}$	3.698	-1.897	9.293
HF	UFP lag0	Main+NO_2+O_3+$PM_{2.5}$	3.677	-1.937	9.291
HF	UFP lag1	Main+NO_2+O_3+$PM_{2.5}$	5.746	-0.972	12.464
HF	UFP lag2	Main+NO_2+O_3+$PM_{2.5}$	-3.880	-9.308	1.547

LF/HF	UFP 1h	Main. reduced	0.013	-0.091	0.117
LF/HF	UFP 3h	Main. reduced	-0.013	-0.115	0.088
LF/HF	UFP 12h	Main. reduced	0.064	-0.038	0.166
LF/HF	UFP 24h	Main. reduced	0.088	-0.036	0.212
LF/HF	UFP lag0	Main. reduced	-0.002	-0.120	0.117
LF/HF	UFP lag1	Main. reduced	0.112	-0.034	0.258
LF/HF	UFP lag2	Main. reduced	0.106	-0.014	0.226
LF/HF	UFP 1h	Main+NO_2	0.010	-0.100	0.120
LF/HF	UFP 3h	Main+NO_2	-0.021	-0.133	0.090
LF/HF	UFP 12h	Main+NO_2	0.070	-0.043	0.182
LF/HF	UFP 24h	Main+NO_2	0.102	-0.036	0.241
LF/HF	UFP lag0	Main+NO_2	-0.018	-0.160	0.125
LF/HF	UFP lag1	Main+NO_2	0.150	-0.017	0.317
LF/HF	UFP lag2	Main+NO_2	0.114	-0.008	0.237
LF/HF	UFP 1h	Main + $PM_{2.5}$	0.067	-0.062	0.196
LF/HF	UFP 3h	Main + $PM_{2.5}$	0.018	-0.102	0.138
LF/HF	UFP 12h	Main + $PM_{2.5}$	0.100	-0.008	0.208
LF/HF	UFP 24h	Main + $PM_{2.5}$	0.153	0.016	0.289
LF/HF	UFP lag0	Main + $PM_{2.5}$	0.096	-0.086	0.278
LF/HF	UFP lag1	Main + $PM_{2.5}$	0.225	0.056	0.394
LF/HF	UFP lag2	Main + $PM_{2.5}$	0.131	0.003	0.259
LF/HF	UFP 1h	Main+O_3	0.017	-0.095	0.130
LF/HF	UFP 3h	Main+O_3	-0.013	-0.122	0.096
LF/HF	UFP 12h	Main+O_3	0.068	-0.036	0.172
LF/HF	UFP 24h	Main+O_3	0.097	-0.031	0.226
LF/HF	UFP lag0	Main+O_3	0.000	-0.133	0.133
LF/HF	UFP lag1	Main+O_3	0.161	-0.007	0.330
LF/HF	UFP lag2	Main+O_3	0.128	0.004	0.252
LF/HF	UFP 1h	Main+NO_2+O_3+$PM_{2.5}$	0.069	-0.061	0.199
LF/HF	UFP 3h	Main+NO_2+O_3+$PM_{2.5}$	0.014	-0.109	0.138
LF/HF	UFP 12h	Main+NO_2+O_3+$PM_{2.5}$	0.097	-0.019	0.213
LF/HF	UFP 24h	Main+NO_2+O_3+$PM_{2.5}$	0.152	0.005	0.299
LF/HF	UFP lag0	Main+NO_2+O_3+$PM_{2.5}$	0.079	-0.120	0.277
LF/HF	UFP lag1	Main+NO_2+O_3+$PM_{2.5}$	0.232	0.048	0.415
LF/HF	UFP lag2	Main+NO_2+O_3+$PM_{2.5}$	0.124	-0.007	0.256

Abbreviations: UFP, ultrafine particles; SDNN, standard deviation of normal to normal intervals; RMSSD, root mean square of successive differences between normal heartbeats; LF, low frequency power; HF, high frequency power; LF/HF, ratio of Low to high frequency power

Supplementary Table 6: Change in Outcomes per IQR exposure increase to UFP, PM_{10} and $PM_{2.5}$ among Children: estimates and 95% CI for both the crude and the main models

Outcome	Exposure	Model	Estimate	CI low	CI high
SDNN	UFP 1h	Crude	-0.765	-2.374	0.845
SDNN	UFP 1h	Main	-0.966	-2.948	1.017
SDNN	UFP 3h	Crude	-1.074	-2.83	0.682
SDNN	UFP 3h	Main	-1.207	-3.541	1.128
SDNN	UFP 12h	Crude	-1.01	-2.649	0.629
SDNN	UFP 12h	Main	-0.472	-3.192	2.247
SDNN	UFP 24h	Crude	-0.507	-1.965	0.95
SDNN	UFP 24h	Main	0.854	-1.849	3.556
SDNN	UFP lag0	Crude	-0.21	-1.601	1.181
SDNN	UFP lag0	Main	-0.326	-2.51	1.857
SDNN	UFP lag1	Crude	0.522	-0.846	1.89
SDNN	UFP lag1	Main	1.331	-0.565	3.228
SDNN	UFP lag2	Crude	0.01	-1.328	1.347
SDNN	UFP lag2	Main	0.164	-2.097	2.424
SDNN	PM_{10} 3h	Crude	-1.169	-2.16	-0.178
SDNN	PM_{10} 3h	Main	-0.608	-1.721	0.505
SDNN	PM_{10} 12h	Crude	-1.159	-2.287	-0.031
SDNN	PM_{10} 12h	Main	-0.619	-1.936	0.698
SDNN	PM_{10} 24h	Crude	-1.004	-2.069	0.06
SDNN	PM_{10} 24h	Main	-0.467	-1.765	0.831
SDNN	PM_{10} lag0	Crude	-0.407	-1.276	0.463
SDNN	PM_{10} lag0	Main	0.067	-0.897	1.03
SDNN	PM_{10} lag1	Crude	-0.426	-1.355	0.504
SDNN	PM_{10} lag1	Main	-0.036	-1.119	1.047
SDNN	PM_{10} lag2	Crude	-0.286	-1.285	0.712
SDNN	PM_{10} lag2	Main	-0.079	-1.128	0.97
SDNN	$PM_{2.5}$ lag0	Crude	-0.167	-1.188	0.855
SDNN	$PM_{2.5}$ lag0	Main	0.09	-1.022	1.203
SDNN	$PM_{2.5}$ lag1	Crude	-0.508	-1.591	0.575
SDNN	$PM_{2.5}$ lag1	Main	-0.471	-1.728	0.787
SDNN	$PM_{2.5}$ lag2	Crude	-0.608	-1.658	0.443
SDNN	$PM_{2.5}$ lag2	Main	-0.579	-1.674	0.516
RMSSD	UFP 1h	Crude	-0.925	-2.797	0.948
RMSSD	UFP 1h	Main	-1.11	-3.324	1.104
RMSSD	UFP 3h	Crude	-1.06	-3.104	0.985
RMSSD	UFP 3h	Main	-0.907	-3.56	1.747
RMSSD	UFP 12h	Crude	-0.788	-2.698	1.122
RMSSD	UFP 12h	Main	0.81	-2.344	3.963
RMSSD	UFP 24h	Crude	-0.467	-2.163	1.23
RMSSD	UFP 24h	Main	1.973	-1.148	5.093
RMSSD	UFP lag0	Crude	0.013	-1.605	1.632
RMSSD	UFP lag0	Main	0.11	-2.188	2.408
RMSSD	UFP lag1	Crude	0.363	-1.23	1.956
RMSSD	UFP lag1	Main	1.255	-0.839	3.349
RMSSD	UFP lag2	Crude	-0.398	-1.954	1.158

RMSSD	UFP lag2	Main	-0.41	-2.653	1.833
RMSSD	PM_{10} 3h	Crude	-1.493	-2.671	-0.314
RMSSD	PM_{10} 3h	Main	-0.911	-2.233	0.412
RMSSD	PM_{10} 12h	Crude	-1.387	-2.729	-0.045
RMSSD	PM_{10} 12h	Main	-0.668	-2.193	0.856
RMSSD	PM_{10} 24h	Crude	-1.219	-2.485	0.048
RMSSD	PM_{10} 24h	Main	-0.504	-1.976	0.969
RMSSD	PM_{10} lag0	Crude	-0.702	-1.736	0.331
RMSSD	PM_{10} lag0	Main	-0.258	-1.392	0.877
RMSSD	PM_{10} lag1	Crude	-0.505	-1.611	0.601
RMSSD	PM_{10} lag1	Main	-0.158	-1.4	1.083
RMSSD	PM_{10} lag2	Crude	-0.406	-1.595	0.782
RMSSD	PM_{10} lag2	Main	-0.365	-1.641	0.912
RMSSD	$PM_{2.5}$ lag0	Crude	-0.511	-1.726	0.705
RMSSD	$PM_{2.5}$ lag0	Main	-0.365	-1.692	0.962
RMSSD	$PM_{2.5}$ lag1	Crude	-0.593	-1.881	0.696
RMSSD	$PM_{2.5}$ lag1	Main	-0.553	-2.001	0.895
RMSSD	$PM_{2.5}$ lag2	Crude	-0.722	-1.972	0.529
RMSSD	$PM_{2.5}$ lag2	Main	-0.889	-2.217	0.44
LF	UFP 1h	Crude	19.695	-40.492	79.883
LF	UFP 1h	Main	-5.408	-73.579	62.763
LF	UFP 3h	Crude	-2.085	-67.815	63.644
LF	UFP 3h	Main	-31.742	-110.235	46.752
LF	UFP 12h	Crude	6.423	-54.952	67.798
LF	UFP 12h	Main	-38.144	-147.69	71.402
LF	UFP 24h	Crude	5.694	-48.797	60.184
LF	UFP 24h	Main	-42.359	-140.391	55.672
LF	UFP lag0	Crude	0.771	-51.196	52.737
LF	UFP lag0	Main	-43.078	-118.901	32.744
LF	UFP lag1	Crude	6.411	-44.755	57.577
LF	UFP lag1	Main	-43.627	-117.256	30.002
LF	UFP lag2	Crude	5.693	-44.287	55.673
LF	UFP lag2	Main	-31.551	-103.103	40
LF	PM_{10} 3h	Crude	-12.692	-49.519	24.136
LF	PM_{10} 3h	Main	-15.926	-56.974	25.121
LF	PM_{10} 12h	Crude	-14.088	-55.961	27.786
LF	PM_{10} 12h	Main	-23.202	-70.886	24.483
LF	PM_{10} 24h	Crude	-17.076	-56.562	22.41
LF	PM_{10} 24h	Main	-22.509	-66.942	21.924
LF	PM_{10} lag0	Crude	-0.526	-32.727	31.676
LF	PM_{10} lag0	Main	-11.598	-48.678	25.482
LF	PM_{10} lag1	Crude	-9.693	-44.119	24.733
LF	PM_{10} lag1	Main	-10.867	-48.601	26.866
LF	PM_{10} lag2	Crude	-19.981	-56.941	16.98
LF	PM_{10} lag2	Main	-30.681	-72.58	11.217
LF	$PM_{2.5}$ lag0	Crude	8.04	-29.787	45.866
LF	$PM_{2.5}$ lag0	Main	-0.996	-43.723	41.731
LF	$PM_{2.5}$ lag1	Crude	-9.192	-49.306	30.921

LF	$PM_{2.5}$ lag1	Main	-8.871	-51.489	33.747
LF	$PM_{2.5}$ lag2	Crude	-37.762	-76.606	1.082
LF	$PM_{2.5}$ lag2	Main	-47.719	-91.12	-4.317
HF	UFP 1h	Crude	-10.073	-60.853	40.707
HF	UFP 1h	Main	-10.623	-72.57	51.325
HF	UFP 3h	Crude	-0.836	-56.178	54.506
HF	UFP 3h	Main	12.884	-59.882	85.651
HF	UFP 12h	Crude	-27.909	-79.667	23.849
HF	UFP 12h	Main	-16.925	-101.851	68.001
HF	UFP 24h	Crude	-28.677	-74.616	17.262
HF	UFP 24h	Main	-5.585	-90.137	78.968
HF	UFP lag0	Crude	-22.536	-66.379	21.307
HF	UFP lag0	Main	-33.359	-101.519	34.801
HF	UFP lag1	Crude	7.822	-35.411	51.054
HF	UFP lag1	Main	40.685	-18.563	99.933
HF	UFP lag2	Crude	5.354	-36.9	47.608
HF	UFP lag2	Main	-23.787	-94.42	46.846
HF	PM_{10} 3h	Crude	-21.908	-53.197	9.38
HF	PM_{10} 3h	Main	-5.99	-41.513	29.533
HF	PM_{10} 12h	Crude	-26.069	-61.642	9.505
HF	PM_{10} 12h	Main	-6.591	-48.619	35.437
HF	PM_{10} 24h	Crude	-22.041	-55.611	11.529
HF	PM_{10} 24h	Main	1.808	-39.61	43.226
HF	PM_{10} lag0	Crude	-6.875	-34.266	20.515
HF	PM_{10} lag0	Main	-0.979	-31.715	29.758
HF	PM_{10} lag1	Crude	-6.322	-35.614	22.969
HF	PM_{10} lag1	Main	14.372	-20.127	48.87
HF	PM_{10} lag2	Crude	4.635	-26.832	36.102
HF	PM_{10} lag2	Main	15.166	-18.252	48.585
HF	$PM_{2.5}$ lag0	Crude	4.767	-27.418	36.951
HF	$PM_{2.5}$ lag0	Main	5.572	-29.918	41.063
HF	$PM_{2.5}$ lag1	Crude	-5.94	-40.067	28.187
HF	$PM_{2.5}$ lag1	Main	11.842	-28.263	51.948
HF	$PM_{2.5}$ lag2	Crude	-8.284	-41.406	24.839
HF	$PM_{2.5}$ lag2	Main	-0.286	-35.232	34.661
LF/HF	UFP 1h	Crude	0.023	-0.122	0.168
LF/HF	UFP 1h	Main	0.066	-0.128	0.261
LF/HF	UFP 3h	Crude	-0.045	-0.198	0.109
LF/HF	UFP 3h	Main	-0.022	-0.244	0.199
LF/HF	UFP 12h	Crude	0.065	-0.077	0.208
LF/HF	UFP 12h	Main	0.244	-0.033	0.521
LF/HF	UFP 24h	Crude	0.106	-0.021	0.234
LF/HF	UFP 24h	Main	0.332	0.093	0.570
LF/HF	UFP lag0	Crude	0.046	-0.084	0.176
LF/HF	UFP lag0	Main	0.106	-0.125	0.337
LF/HF	UFP lag1	Crude	0.044	-0.080	0.168
LF/HF	UFP lag1	Main	0.126	-0.080	0.331
LF/HF	UFP lag2	Crude	0.025	-0.093	0.143

LF/HF	UFP lag2	Main	0.101	-0.121	0.322
LF/HF	PM_{10} 3h	Crude	0.004	0.117	0.125
LF/HF	PM_{10} 3h	Main	0.062	-0.107	0.232
LF/HF	PM_{10} 12h	Crude	-0.008	-0.158	0.142
LF/HF	PM_{10} 12h	Main	0.056	-0.180	0.291
LF/HF	PM_{10} 24h	Crude	-0.013	-0.153	0.127
LF/HF	PM_{10} 24h	Main	0.027	-0.190	0.243
LF/HF	PM_{10} lag0	Crude	-0.061	-0.169	0.048
LF/HF	PM_{10} lag0	Main	-0.056	-0.205	0.093
LF/HF	PM_{10} lag1	Crude	-0.046	-0.159	0.068
LF/HF	PM_{10} lag1	Main	-0.076	-0.259	0.107
LF/HF	PM_{10} lag2	Crude	-0.107	-0.241	-0.028
LF/HF	PM_{10} lag2	Main	-0.166	-0.342	0.011
LF/HF	$PM_{2.5}$ lag0	Crude	-0.051	-0.169	0.067
LF/HF	$PM_{2.5}$ lag0	Main	-0.018	-0.186	0.150
LF/HF	$PM_{2.5}$ lag1	Crude	-0.005	-0.150	0.139
LF/HF	$PM_{2.5}$ lag1	Main	0.019	-0.197	0.235
LF/HF	$PM_{2.5}$ lag2	Crude	-0.136	-0.293	0.020
LF/HF	$PM_{2.5}$ lag2	Main	-0.212	-0.409	-0.015

Abbreviations: UFP, ultrafine particles; PM, particulate matter; SDNN, standard deviation of normal to normal intervals; RMSSD, root mean square of successive differences between normal heartbeats; LF, low frequency power; HF, high frequency power; LF/HF, ratio of Low to high frequency power

Supplementary Table 7: Change in Outcomes per IQR exposure increase to UFP among Children: estimates and 95% CI for two and multi-pollutant models

**Outcome**	**Exposure**	**Model**	**Estimate**	**CI low**	**CI high**
SDNN	UFP 1h	Main. reduced	-0.731	-3.247	1.785
SDNN	UFP 3h	Main. reduced	-0.982	-4.028	2.063
SDNN	UFP 12h	Main. reduced	1.535	-2.770	5.840
SDNN	UFP 24h	Main. reduced	1.545	-2.160	5.250
SDNN	UFP lag0	Main. reduced	-0.965	-4.122	2.192
SDNN	UFP lag1	Main. reduced	0.608	-1.673	2.890
SDNN	UFP lag2	Main. reduced	0.330	-3.483	4.143
SDNN	UFP 1h	Main+NO_2	-0.881	-3.391	1.628
SDNN	UFP 3h	Main+NO_2	-0.966	-3.999	2.067
SDNN	UFP 12h	Main+NO_2	3.511	-1.132	8.154
SDNN	UFP 24h	Main+NO_2	2.631	-1.201	6.464
SDNN	UFP lag0	Main+NO_2	-0.273	-3.537	2.992
SDNN	UFP lag1	Main+NO_2	0.650	-1.621	2.922
SDNN	UFP lag2	Main+NO_2	-1.514	-5.805	2.776
SDNN	UFP 1h	Main + $PM_{2.5}$	-0.911	-3.449	1.627
SDNN	UFP 3h	Main + $PM_{2.5}$	-1.116	-4.172	1.940
SDNN	UFP 12h	Main + $PM_{2.5}$	2.049	-2.349	6.447
SDNN	UFP 24h	Main + $PM_{2.5}$	1.878	-1.875	5.631
SDNN	UFP lag0	Main + $PM_{2.5}$	-1.066	-4.229	2.098
SDNN	UFP lag1	Main + $PM_{2.5}$	0.731	-1.563	3.025
SDNN	UFP lag2	Main + $PM_{2.5}$	-0.421	-4.543	3.700
SDNN	UFP 1h	Main+O_3	-0.592	-3.147	1.963
SDNN	UFP 3h	Main+O_3	-0.736	-3.890	2.418
SDNN	UFP 12h	Main+O_3	2.642	-2.080	7.364
SDNN	UFP 24h	Main+O_3	2.128	-1.745	6.002
SDNN	UFP lag0	Main+O_3	-0.737	-3.984	2.510
SDNN	UFP lag1	Main+O_3	0.610	-1.674	2.894
SDNN	UFP lag2	Main+O_3	-0.368	-4.625	3.888
SDNN	UFP 1h	Main+NO_2+O_3+$PM_{2.5}$	-0.833	-3.445	1.778
SDNN	UFP 3h	Main+NO_2+O_3+$PM_{2.5}$	-0.880	-4.098	2.339
SDNN	UFP 12h	Main+NO_2+O_3+$PM_{2.5}$	4.544	-0.502	9.591
SDNN	UFP 24h	Main+NO_2+O_3+$PM_{2.5}$	3.032	-0.950	7.014
SDNN	UFP lag0	Main+NO_2+O_3+$PM_{2.5}$	-0.158	-3.680	3.363
SDNN	UFP lag1	Main+NO_2+O_3+$PM_{2.5}$	0.654	-1.645	2.954
SDNN	UFP lag2	Main+NO_2+O_3+$PM_{2.5}$	-2.229	-6.943	2.485
RMSSD	UFP 1h	Main. reduced	-0.161	-2.955	2.632
RMSSD	UFP 3h	Main. reduced	-0.461	-3.836	2.914
RMSSD	UFP 12h	Main. reduced	2.358	-2.528	7.244
RMSSD	UFP 24h	Main. reduced	2.430	-1.808	6.669
RMSSD	UFP lag0	Main. reduced	0.157	-2.732	3.047
RMSSD	UFP lag1	Main. reduced	1.233	-1.403	3.870
RMSSD	UFP lag2	Main. reduced	0.813	-2.719	4.346
RMSSD	UFP 1h	Main+NO_2	-0.173	-2.963	2.617
RMSSD	UFP 3h	Main+NO_2	-0.334	-3.711	3.043

RMSSD	UFP 12h	Main+NO_2	4.417	-0.923	9.757
RMSSD	UFP 24h	Main+NO_2	3.611	-0.823	8.045
RMSSD	UFP lag0	Main+NO_2	0.794	-2.234	3.822
RMSSD	UFP lag1	Main+NO_2	1.337	-1.299	3.973
RMSSD	UFP lag2	Main+NO_2	0.250	-3.401	3.901
RMSSD	UFP 1h	Main + $PM_{2.5}$	-0.302	-3.150	2.546
RMSSD	UFP 3h	Main + $PM_{2.5}$	-0.570	-3.976	2.836
RMSSD	UFP 12h	Main + $PM_{2.5}$	2.700	-2.287	7.687
RMSSD	UFP 24h	Main + $PM_{2.5}$	2.672	-1.628	6.973
RMSSD	UFP lag0	Main + $PM_{2.5}$	0.070	-2.847	2.987
RMSSD	UFP lag1	Main + $PM_{2.5}$	1.275	-1.371	3.921
RMSSD	UFP lag2	Main + $PM_{2.5}$	0.532	-3.354	4.417
RMSSD	UFP 1h	Main+O_3	-0.047	-2.851	2.758
RMSSD	UFP 3h	Main+O_3	-0.199	-3.619	3.222
RMSSD	UFP 12h	Main+O_3	3.501	-1.650	8.652
RMSSD	UFP 24h	Main+O_3	2.987	-1.337	7.312
RMSSD	UFP lag0	Main+O_3	0.202	-2.689	3.093
RMSSD	UFP lag1	Main+O_3	1.190	-1.449	3.828
RMSSD	UFP lag2	Main+O_3	-0.017	-4.028	3.995
RMSSD	UFP 1h	Main+NO_2+O_3+$PM_{2.5}$	0.101	-2.808	3.011
RMSSD	UFP 3h	Main+NO_2+O_3+$PM_{2.5}$	0.079	-3.461	3.619
RMSSD	UFP 12h	Main+NO_2+O_3+$PM_{2.5}$	5.779	0.152	11.405
RMSSD	UFP 24h	Main+NO_2+O_3+$PM_{2.5}$	4.099	-0.413	8.611
RMSSD	UFP lag0	Main+NO_2+O_3+$PM_{2.5}$	1.162	-2.076	4.400
RMSSD	UFP lag1	Main+NO_2+O_3+$PM_{2.5}$	1.264	-1.388	3.916
RMSSD	UFP lag2	Main+NO_2+O_3+$PM_{2.5}$	-0.329	-4.527	3.870
LF	UFP 1h	Main. reduced	30.59	-60.779	121.96
LF	UFP 3h	Main. reduced	-8.85	-114.743	97.043
LF	UFP 12h	Main. reduced	-37.361	-189.204	114.481
LF	UFP 24h	Main. reduced	-27.727	-152.723	97.269
LF	UFP lag0	Main. reduced	-70.591	-209.616	68.435
LF	UFP lag1	Main. reduced	39.717	-59.394	138.829
LF	UFP lag2	Main. reduced	55.866	-43.433	155.165
LF	UFP 1h	Main+NO_2	31.682	-60.037	123.4
LF	UFP 3h	Main+NO_2	-7.942	-114.176	98.291
LF	UFP 12h	Main+NO_2	-39.812	-192.458	112.834
LF	UFP 24h	Main+NO_2	-27.981	-153.282	97.321
LF	UFP lag0	Main+NO_2	-91.209	-239.913	57.494
LF	UFP lag1	Main+NO_2	37.652	-63.053	138.357
LF	UFP lag2	Main+NO_2	65.236	-37.763	168.235
LF	UFP 1h	Main + $PM_{2.5}$	38.18	-54.116	130.477
LF	UFP 3h	Main + $PM_{2.5}$	2.894	-105.551	111.339
LF	UFP 12h	Main + $PM_{2.5}$	-31.709	-183.974	120.555
LF	UFP 24h	Main + $PM_{2.5}$	-36.469	-162.408	89.47
LF	UFP lag0	Main + $PM_{2.5}$	-64.369	-204.053	75.314
LF	UFP lag1	Main + $PM_{2.5}$	31.013	-70.11	132.135
LF	UFP lag2	Main + $PM_{2.5}$	44.016	-61.191	149.223
LF	UFP 1h	Main+O_3	35.494	-55.892	126.880

LF	UFP 3h	Main+O_3	-6.635	-112.351	99.082
LF	UFP 12h	Main+O_3	-8.806	-166.148	148.536
LF	UFP 24h	Main+O_3	7.893	-127.211	142.996
LF	UFP lag0	Main+O_3	-42.272	-189.376	104.832
LF	UFP lag1	Main+O_3	69.550	-34.482	173.582
LF	UFP lag2	Main+O_3	57.832	-41.278	156.941
LF	UFP 1h	Main+NO_2+O_3+$PM_{2.5}$	46.236	-46.474	138.947
LF	UFP 3h	Main+NO_2+O_3+$PM_{2.5}$	8.155	-100.498	116.808
LF	UFP 12h	Main+NO_2+O_3+$PM_{2.5}$	-3.479	-161.773	154.815
LF	UFP 24h	Main+NO_2+O_3+$PM_{2.5}$	-0.030	-136.338	136.279
LF	UFP lag0	Main+NO_2+O_3+$PM_{2.5}$	-54.916	-211.994	102.163
LF	UFP lag1	Main+NO_2+O_3+$PM_{2.5}$	57.211	-50.424	164.846
LF	UFP lag2	Main+NO_2+O_3+$PM_{2.5}$	57.267	-51.358	165.891
HF	UFP 1h	Main. reduced	-4.944	-84.939	75.052
HF	UFP 3h	Main. reduced	14.746	-81.801	111.294
HF	UFP 12h	Main. reduced	-39.631	-176.672	97.409
HF	UFP 24h	Main. reduced	-46.332	-164.371	71.707
HF	UFP lag0	Main. reduced	-70.215	-170.434	30.004
HF	UFP lag1	Main. reduced	2.418	-70.332	75.168
HF	UFP lag2	Main. reduced	-5.206	-126.801	116.389
HF	UFP 1h	Main+NO_2	-13.071	-91.731	65.589
HF	UFP 3h	Main+NO_2	14.536	-80.290	109.362
HF	UFP 12h	Main+NO_2	43.214	-103.081	189.510
HF	UFP 24h	Main+NO_2	0.647	-120.199	121.494
HF	UFP lag0	Main+NO_2	-34.650	-137.058	67.759
HF	UFP lag1	Main+NO_2	4.677	-66.707	76.062
HF	UFP lag2	Main+NO_2	-110.247	-244.029	23.536
HF	UFP 1h	Main + $PM_{2.5}$	-11.888	-92.474	68.697
HF	UFP 3h	Main + $PM_{2.5}$	9.603	-87.162	106.368
HF	UFP 12h	Main + $PM_{2.5}$	-22.767	-162.759	117.225
HF	UFP 24h	Main + $PM_{2.5}$	-35.305	-154.84	84.231
HF	UFP lag0	Main + $PM_{2.5}$	-74.509	-174.74	25.721
HF	UFP lag1	Main + $PM_{2.5}$	7.316	-65.746	80.379
HF	UFP lag2	Main + $PM_{2.5}$	-39.902	-170.946	91.141
HF	UFP 1h	Main+O_3	2.981	-78.082	84.044
HF	UFP 3h	Main+O_3	31.338	-68.283	130.959
HF	UFP 12h	Main+O_3	-7.473	-157.958	143.011
HF	UFP 24h	Main+O_3	-28.335	-151.813	95.143
HF	UFP lag0	Main+O_3	-59.763	-162.772	43.245
HF	UFP lag1	Main+O_3	2.521	-70.16	75.201
HF	UFP lag2	Main+O_3	-46.062	-181.328	89.204
HF	UFP 1h	Main+NO_2+O_3+$PM_{2.5}$	-5.609	-87.38	76.162
HF	UFP 3h	Main+NO_2+O_3+$PM_{2.5}$	29.684	-70.682	130.051
HF	UFP 12h	Main+NO_2+O_3+$PM_{2.5}$	75.174	-83.906	234.254
HF	UFP 24h	Main+NO_2+O_3+$PM_{2.5}$	12.623	-112.964	138.211
HF	UFP lag0	Main+NO_2+O_3+$PM_{2.5}$	-24.832	-135.284	85.621
HF	UFP lag1	Main+NO_2+O_3+$PM_{2.5}$	2.815	-69.354	74.983
HF	UFP lag2	Main+NO_2+O_3+$PM_{2.5}$	-150.217	-296.588	-3.845

LF/HF	UFP 1h	Main. reduced	0.078	-0.115	0.271
LF/HF	UFP 3h	Main. reduced	-0.030	-0.249	0.190
LF/HF	UFP 12h	Main. reduced	0.171	-0.125	0.466
LF/HF	UFP 24h	Main. reduced	0.286	0.034	0.539
LF/HF	UFP lag0	Main. reduced	0.059	-0.177	0.294
LF/HF	UFP lag1	Main. reduced	0.120	-0.084	0.325
LF/HF	UFP lag2	Main. reduced	0.124	-0.097	0.345
LF/HF	UFP 1h	Main+NO_2	0.066	-0.128	0.260
LF/HF	UFP 3h	Main+NO_2	-0.036	-0.258	0.186
LF/HF	UFP 12h	Main+NO_2	0.210	-0.083	0.503
LF/HF	UFP 24h	Main+NO_2	0.354	0.069	0.639
LF/HF	UFP lag0	Main+NO_2	0.065	-0.180	0.311
LF/HF	UFP lag1	Main+NO_2	0.082	-0.155	0.319
LF/HF	UFP lag2	Main+NO_2	0.090	-0.132	0.312
LF/HF	UFP 1h	Main + $PM_{2.5}$	0.050	-0.099	0.199
LF/HF	UFP 3h	Main + $PM_{2.5}$	-0.005	-0.166	0.156
LF/HF	UFP 12h	Main + $PM_{2.5}$	0.099	-0.052	0.249
LF/HF	UFP 24h	Main + $PM_{2.5}$	0.124	-0.010	0.258
LF/HF	UFP lag0	Main + $PM_{2.5}$	0.050	-0.084	0.184
LF/HF	UFP lag1	Main + $PM_{2.5}$	0.020	-0.108	0.149
LF/HF	UFP lag2	Main + $PM_{2.5}$	0.036	-0.086	0.159
LF/HF	UFP 1h	Main+O_3	0.079	-0.118	0.277
LF/HF	UFP 3h	Main+O_3	-0.012	-0.237	0.213
LF/HF	UFP 12h	Main+O_3	0.247	-0.031	0.525
LF/HF	UFP 24h	Main+O_3	0.353	0.111	0.595
LF/HF	UFP lag0	Main+O_3	0.166	-0.090	0.423
LF/HF	UFP lag1	Main+O_3	0.164	-0.054	0.382
LF/HF	UFP lag2	Main+O_3	0.203	-0.066	0.471
LF/HF	UFP 1h	Main+NO_2+O_3+$PM_{2.5}$	0.095	-0.102	0.292
LF/HF	UFP 3h	Main+NO_2+O_3+$PM_{2.5}$	-0.025	-0.248	0.198
LF/HF	UFP 12h	Main+NO_2+O_3+$PM_{2.5}$	0.142	-0.166	0.450
LF/HF	UFP 24h	Main+NO_2+O_3+$PM_{2.5}$	0.318	0.023	0.613
LF/HF	UFP lag0	Main+NO_2+O_3+$PM_{2.5}$	0.087	-0.182	0.356
LF/HF	UFP lag1	Main+NO_2+O_3+$PM_{2.5}$	0.135	-0.109	0.378
LF/HF	UFP lag2	Main+NO_2+O_3+$PM_{2.5}$	0.250	-0.019	0.518

Abbreviations: UFP, ultrafine particles; SDNN, standard deviation of normal to normal intervals; RMSSD, root mean square of successive differences between normal heartbeats; LF, low frequency power; HF, high frequency power; LF/HF, ratio of Low to high frequency power

Supplementary Figure 3: Change in Outcomes per IQR exposure increase to PM10 among Seniors – Two and Multi-Pollutant Models

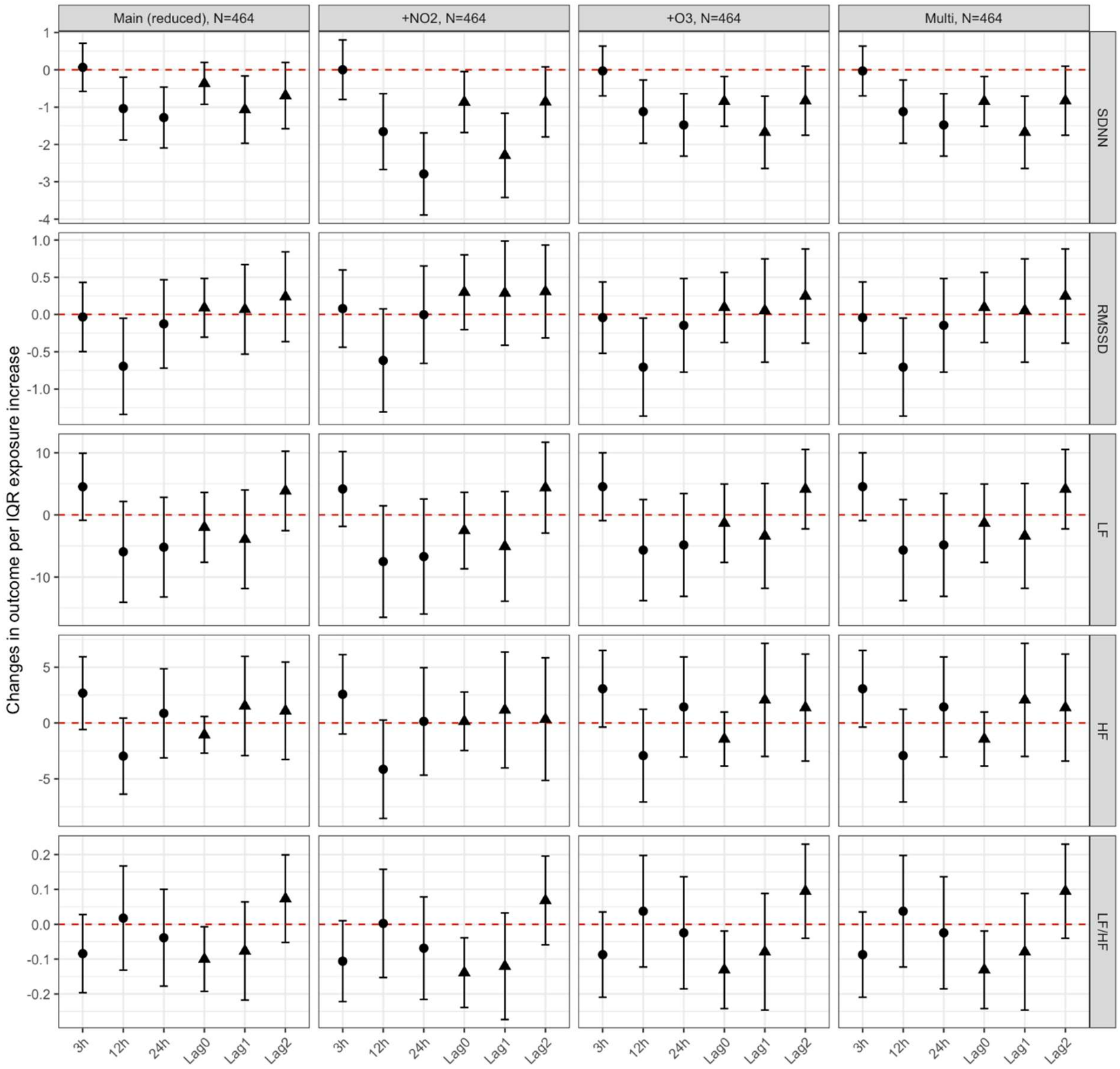

Abbreviations: PM, particulate matter; SDNN, standard deviation of normal to normal intervals; RMSSD, root mean square of successive differences between normal heartbeats; LF, low frequency power; HF, high frequency power; LF/HF, ratio of Low to high frequency power

Supplementary Table 8: Change in Outcomes per IQR exposure increase to PM10 among Seniors – Two and Multi-Pollutant Models – Estimates and 95% CI

Outcome	Exposure	Model	Estimate	CI low	CI high
SDNN	PM_{10}_3h	Main, reduced	0.066	-0.581	0.713
SDNN	PM_{10} 12h	Main, reduced	-1.038	-1.880	-0.196
SDNN	PM_{10} 24h	Main, reduced	-1.279	-2.094	-0.464
SDNN	PM_{10} lag0	Main, reduced	-0.363	-0.926	0.199
SDNN	PM_{10} lag1	Main, reduced	-1.067	-1.967	-0.166
SDNN	PM_{10} lag2	Main, reduced	-0.690	-1.579	0.198
SDNN	PM_{10}_3h	Main+NO_2	0.002	-0.797	0.801
SDNN	PM_{10} 12h	Main+NO_2	-1.655	-2.672	-0.638
SDNN	PM_{10} 24h	Main+NO_2	-2.790	-3.889	-1.692
SDNN	PM_{10} lag0	Main+NO_2	-0.863	-1.679	-0.047
SDNN	PM_{10} lag1	Main+NO_2	-2.291	-3.419	-1.162
SDNN	PM_{10} lag2	Main+NO_2	-0.859	-1.797	0.080
SDNN	PM_{10}_3h	Main+O_3	-0.031	-0.698	0.636
SDNN	PM_{10} 12h	Main+O_3	-1.120	-1.966	-0.274
SDNN	PM_{10} 24h	Main+O_3	-1.476	-2.311	-0.640
SDNN	PM_{10} lag0	Main+O_3	-0.846	-1.511	-0.180
SDNN	PM_{10} lag1	Main+O_3	-1.673	-2.642	-0.704
SDNN	PM_{10} lag2	Main+O_3	-0.829	-1.753	0.096
SDNN	PM_{10}_3h	Main+NO_2+O_3	0.020	-0.782	0.822
SDNN	PM_{10} 12h	Main+NO_2+O_3	-1.613	-2.651	-0.576
SDNN	PM_{10} 24h	Main+NO_2+O_3	-2.789	-3.908	-1.673
SDNN	PM_{10} lag0	Main+NO_2+O_3	-1.197	-2.045	-0.349
SDNN	PM_{10} lag1	Main+NO_2+O_3	-2.613	-3.693	-1.523
SDNN	PM_{10} lag2	Main+NO_2+O_3	-0.807	-1.823	0.082
RMSSD	PM_{10}_3h	Main, reduced	-0.033	-0.497	0.431
RMSSD	PM_{10} 12h	Main, reduced	-0.695	-1.340	-0.050
RMSSD	PM_{10} 24h	Main, reduced	-0.126	-0.719	0.467
RMSSD	PM_{10} lag0	Main, reduced	0.090	-0.305	0.484
RMSSD	PM_{10} lag1	Main, reduced	0.069	-0.532	0.671
RMSSD	PM_{10} lag2	Main, reduced	0.238	-0.364	0.841
RMSSD	PM_{10}_3h	Main+NO_2	0.080	-0.439	0.598
RMSSD	PM_{10} 12h	Main+NO_2	-0.616	-1.308	0.076
RMSSD	PM_{10} 24h	Main+NO_2	-0.002	-0.657	0.652
RMSSD	PM_{10} lag0	Main+NO_2	0.299	-0.203	0.801
RMSSD	PM_{10} lag1	Main+NO_2	0.287	-0.411	0.986
RMSSD	PM_{10} lag2	Main+NO_2	0.309	-0.315	0.932
RMSSD	PM_{10}_3h	Main+O_3	-0.042	-0.520	0.437
RMSSD	PM_{10} 12h	Main+O_3	-0.706	-1.364	-0.048
RMSSD	PM_{10} 24h	Main+O_3	-0.146	-0.774	0.482
RMSSD	PM_{10} lag0	Main+O_3	0.095	-0.375	0.565
RMSSD	PM_{10} lag1	Main+O_3	0.054	-0.639	0.747

RMSSD	PM_{10} lag2	Main+O_3	0.248	-0.384	0.880
RMSSD	PM_{10}_3h	Main+NO_2+O_3	0.143	-0.384	0.669
RMSSD	PM_{10} 12h	Main+NO_2+O_3	-0.519	-1.219	0.182
RMSSD	PM_{10} 24h	Main+NO_2+O_3	-0.058	-0.709	0.594
RMSSD	PM_{10} lag0	Main+NO_2+O_3	0.226	-0.288	0.740
RMSSD	PM_{10} lag1	Main+NO_2+O_3	0.099	-0.616	0.814
RMSSD	PM_{10} lag2	Main+NO_2+O_3	0.184	-0.454	0.823
LF	PM_{10}_3h	Main, reduced	4.529	-0.869	9.928
LF	PM_{10} 12h	Main, reduced	-5.948	-14.061	2.165
LF	PM_{10} 24h	Main, reduced	-5.203	-13.229	2.823
LF	PM_{10} lag0	Main, reduced	-1.997	-7.622	3.628
LF	PM_{10} lag1	Main, reduced	-3.923	-11.865	4.019
LF	PM_{10} lag2	Main, reduced	3.856	-2.531	10.243
LF	PM_{10}_3h	Main+NO_2	4.171	-1.848	10.191
LF	PM_{10} 12h	Main+NO_2	-7.518	-16.492	1.455
LF	PM_{10} 24h	Main+NO_2	-6.709	-15.975	2.557
LF	PM_{10} lag0	Main+NO_2	-2.518	-8.680	3.644
LF	PM_{10} lag1	Main+NO_2	-5.076	-13.909	3.756
LF	PM_{10} lag2	Main+NO_2	4.382	-2.935	11.698
LF	PM_{10}_3h	Main+O_3	4.541	-0.919	10.002
LF	PM_{10} 12h	Main+O_3	-5.670	-13.810	2.469
LF	PM_{10} 24h	Main+O_3	-4.845	-13.115	3.425
LF	PM_{10} lag0	Main+O_3	-1.336	-7.643	4.970
LF	PM_{10} lag1	Main+O_3	-3.385	-11.829	5.060
LF	PM_{10} lag2	Main+O_3	4.135	-2.256	10.527
LF	PM_{10}_3h	Main+NO_2+O_3	4.263	-1.816	10.342
LF	PM_{10} 12h	Main+NO_2+O_3	-7.304	-16.271	1.663
LF	PM_{10} 24h	Main+NO_2+O_3	-6.349	-15.734	3.036
LF	PM_{10} lag0	Main+NO_2+O_3	-1.881	-8.625	4.863
LF	PM_{10} lag1	Main+NO_2+O_3	-4.494	-13.694	4.706
LF	PM_{10} lag2	Main+NO_2+O_3	4.665	-2.606	11.937
HF	PM_{10}_3h	Main, reduced	2.675	-0.592	5.942
HF	PM_{10} 12h	Main, reduced	-2.968	-6.374	0.438
HF	PM_{10} 24h	Main, reduced	0.865	-3.126	4.855
HF	PM_{10} lag0	Main, reduced	-1.062	-2.703	0.579
HF	PM_{10} lag1	Main, reduced	1.523	-2.922	5.969
HF	PM_{10} lag2	Main, reduced	1.090	-3.275	5.455
HF	PM_{10}_3h	Main+NO_2	2.571	-0.984	6.126
HF	PM_{10} 12h	Main+NO_2	-4.144	-8.554	0.265
HF	PM_{10} 24h	Main+NO_2	0.142	-4.673	4.957
HF	PM_{10} lag0	Main+NO_2	0.149	-2.473	2.771
HF	PM_{10} lag1	Main+NO_2	1.165	-4.026	6.355
HF	PM_{10} lag2	Main+NO_2	0.342	-5.147	5.832
HF	PM_{10}_3h	Main+O_3	3.066	-0.372	6.505

HF	PM_{10} 12h	Main+O_3	-2.924	-7.075	1.228
HF	PM_{10} 24h	Main+O_3	1.440	-3.048	5.927
HF	PM_{10} lag0	Main+O_3	-1.434	-3.851	0.983
HF	PM_{10} lag1	Main+O_3	2.065	-3.002	7.132
HF	PM_{10} lag2	Main+O_3	1.377	-3.419	6.172
HF	PM_{10}_3h	Main+NO_2+O_3	2.920	-0.694	6.534
HF	PM_{10} 12h	Main+NO_2+O_3	-3.850	-8.741	1.040
HF	PM_{10} 24h	Main+NO_2+O_3	1.032	-4.073	6.137
HF	PM_{10} lag0	Main+NO_2+O_3	0.900	-2.281	4.082
HF	PM_{10} lag1	Main+NO_2+O_3	1.892	-3.630	7.414
HF	PM_{10} lag2	Main+NO_2+O_3	0.328	-5.335	5.991
LF/HF	PM_{10}_3h	Main, reduced	-0.084	-0.196	0.028
LF/HF	PM_{10} 12h	Main, reduced	0.018	-0.132	0.167
LF/HF	PM_{10} 24h	Main, reduced	-0.039	-0.177	0.100
LF/HF	PM_{10} lag0	Main, reduced	-0.100	-0.193	-0.007
LF/HF	PM_{10} lag1	Main, reduced	-0.077	-0.217	0.064
LF/HF	PM_{10} lag2	Main, reduced	0.073	-0.052	0.199
LF/HF	PM_{10}_3h	Main+NO_2	-0.106	-0.222	0.010
LF/HF	PM_{10} 12h	Main+NO_2	0.002	-0.153	0.158
LF/HF	PM_{10} 24h	Main+NO_2	-0.069	-0.216	0.079
LF/HF	PM_{10} lag0	Main+NO_2	-0.139	-0.239	-0.039
LF/HF	PM_{10} lag1	Main+NO_2	-0.120	-0.273	0.032
LF/HF	PM_{10} lag2	Main+NO_2	0.068	-0.059	0.196
LF/HF	PM_{10}_3h	Main+O_3	-0.087	-0.210	0.035
LF/HF	PM_{10} 12h	Main+O_3	0.037	-0.123	0.197
LF/HF	PM_{10} 24h	Main+O_3	-0.025	-0.185	0.136
LF/HF	PM_{10} lag0	Main+O_3	-0.131	-0.242	-0.019
LF/HF	PM_{10} lag1	Main+O_3	-0.079	-0.246	0.088
LF/HF	PM_{10} lag2	Main+O_3	0.095	-0.040	0.230
LF/HF	PM_{10}_3h	Main+NO_2+O_3	-0.084	-0.206	0.039
LF/HF	PM_{10} 12h	Main+NO_2+O_3	0.043	-0.116	0.201
LF/HF	PM_{10} 24h	Main+NO_2+O_3	-0.016	-0.175	0.143
LF/HF	PM_{10} lag0	Main+NO_2+O_3	-0.126	-0.239	-0.013
LF/HF	PM_{10} lag1	Main+NO_2+O_3	-0.082	-0.250	0.086
LF/HF	PM_{10} lag2	Main+NO_2+O_3	0.132	-0.009	0.272

Supplementary Figure 4: Change in Outcomes per IQR exposure increase to PM10 among Children – Two and Multi-Pollutant Models

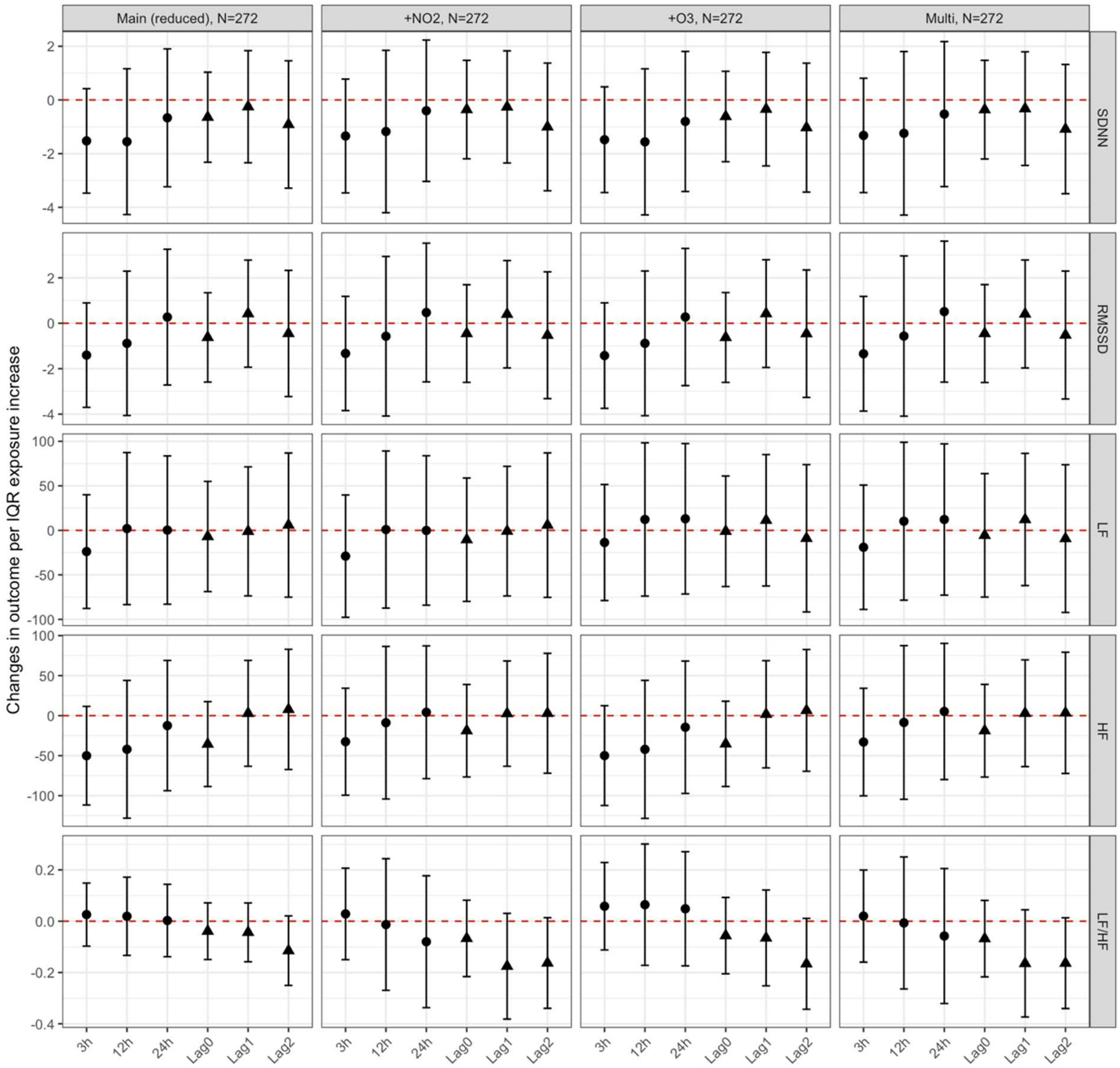

Abbreviations: PM, particulate matter; SDNN, standard deviation of normal to normal intervals; RMSSD, root mean square of successive differences between normal heartbeats; LF, low frequency power; HF, high frequency power; LF/HF, ratio of Low to high frequency power

Supplementary Table 9: Change in Outcomes per IQR exposure increase to PM10 among Children – Two and Multi-Pollutant Models – Estimates and 95% CI

**Outcome**	**Exposure**	**Model**	**Estimate**	**CI low**	**CI high**
SDNN	PM_{10}_3h	Main, reduced	-1.525	-3.470	0.421
SDNN	PM_{10} 12h	Main, reduced	-1.553	-4.268	1.162
SDNN	PM_{10} 24h	Main, reduced	-0.665	-3.233	1.903
SDNN	PM_{10} lag0	Main, reduced	-0.642	-2.320	1.035
SDNN	PM_{10} lag1	Main, reduced	-0.252	-2.340	1.836
SDNN	PM_{10} lag2	Main, reduced	-0.914	-3.286	1.459
SDNN	PM_{10}_3h	Main+NO_2	-1.343	-3.462	0.777
SDNN	PM_{10} 12h	Main+NO_2	-1.176	-4.199	1.848
SDNN	PM_{10} 24h	Main+NO_2	-0.402	-3.035	2.231
SDNN	PM_{10} lag0	Main+NO_2	-0.359	-2.192	1.475
SDNN	PM_{10} lag1	Main+NO_2	-0.260	-2.349	1.829
SDNN	PM_{10} lag2	Main+NO_2	-1.005	-3.384	1.374
SDNN	PM_{10}_3h	Main+O_3	-1.480	-3.450	0.489
SDNN	PM_{10} 12h	Main+O_3	-1.560	-4.280	1.159
SDNN	PM_{10} 24h	Main+O_3	-0.801	-3.409	1.807
SDNN	PM_{10} lag0	Main+O_3	-0.616	-2.300	1.068
SDNN	PM_{10} lag1	Main+O_3	-0.342	-2.456	1.772
SDNN	PM_{10} lag2	Main+O_3	-1.030	-3.433	1.372
SDNN	PM_{10}_3h	Main+NO_2+O_3	-1.321	-3.452	0.809
SDNN	PM_{10} 12h	Main+NO_2+O_3	-1.243	-4.288	1.802
SDNN	PM_{10} 24h	Main+NO_2+O_3	-0.527	-3.227	2.174
SDNN	PM_{10} lag0	Main+NO_2+O_3	-0.362	-2.200	1.476
SDNN	PM_{10} lag1	Main+NO_2+O_3	-0.323	-2.439	1.793
SDNN	PM_{10} lag2	Main+NO_2+O_3	-1.084	-3.490	1.322
RMSSD	PM_{10}_3h	Main, reduced	-1.403	-3.703	0.896
RMSSD	PM_{10} 12h	Main, reduced	-0.884	-4.062	2.293
RMSSD	PM_{10} 24h	Main, reduced	0.271	-2.717	3.258
RMSSD	PM_{10} lag0	Main, reduced	-0.623	-2.590	1.345
RMSSD	PM_{10} lag1	Main, reduced	0.422	-1.934	2.779
RMSSD	PM_{10} lag2	Main, reduced	-0.450	-3.227	2.327
RMSSD	PM_{10}_3h	Main+NO_2	-1.330	-3.845	1.184
RMSSD	PM_{10} 12h	Main+NO_2	-0.572	-4.084	2.940
RMSSD	PM_{10} 24h	Main+NO_2	0.472	-2.581	3.525
RMSSD	PM_{10} lag0	Main+NO_2	-0.452	-2.601	1.696
RMSSD	PM_{10} lag1	Main+NO_2	0.397	-1.965	2.759
RMSSD	PM_{10} lag2	Main+NO_2	-0.526	-3.317	2.265
RMSSD	PM_{10}_3h	Main+O_3	-1.424	-3.745	0.898
RMSSD	PM_{10} 12h	Main+O_3	-0.885	-4.071	2.301
RMSSD	PM_{10} 24h	Main+O_3	0.274	-2.745	3.294
RMSSD	PM_{10} lag0	Main+O_3	-0.625	-2.602	1.351
RMSSD	PM_{10} lag1	Main+O_3	0.424	-1.946	2.795

RMSSD	PM_{10} lag2	Main+O_3	-0.459	-3.264	2.346
RMSSD	PM_{10}_3h	Main+NO_2+O_3	-1.343	-3.868	1.182
RMSSD	PM_{10} 12h	Main+NO_2+O_3	-0.564	-4.091	2.963
RMSSD	PM_{10} 24h	Main+NO_2+O_3	0.511	-2.592	3.614
RMSSD	PM_{10} lag0	Main+NO_2+O_3	-0.453	-2.606	1.701
RMSSD	PM_{10} lag1	Main+NO_2+O_3	0.408	-1.967	2.784
RMSSD	PM_{10} lag2	Main+NO_2+O_3	-0.518	-3.334	2.297
LF	PM_{10}_3h	Main, reduced	-23.855	-87.736	40.026
LF	PM_{10} 12h	Main, reduced	1.963	-83.460	87.387
LF	PM_{10} 24h	Main, reduced	0.288	-83.014	83.589
LF	PM_{10} lag0	Main, reduced	-6.921	-68.782	54.939
LF	PM_{10} lag1	Main, reduced	-1.153	-73.632	71.327
LF	PM_{10} lag2	Main, reduced	5.890	-75.075	86.855
LF	PM_{10}_3h	Main+NO_2	-28.974	-97.700	39.751
LF	PM_{10} 12h	Main+NO_2	0.899	-87.350	89.148
LF	PM_{10} 24h	Main+NO_2	-0.162	-84.086	83.761
LF	PM_{10} lag0	Main+NO_2	-10.516	-79.822	58.789
LF	PM_{10} lag1	Main+NO_2	-0.908	-73.708	71.893
LF	PM_{10} lag2	Main+NO_2	5.853	-75.304	87.010
LF	PM_{10}_3h	Main+O_3	-13.670	-78.863	51.523
LF	PM_{10} 12h	Main+O_3	12.218	-73.862	98.297
LF	PM_{10} 24h	Main+O_3	13.020	-71.458	97.499
LF	PM_{10} lag0	Main+O_3	-1.031	-63.162	61.099
LF	PM_{10} lag1	Main+O_3	11.305	-62.520	85.130
LF	PM_{10} lag2	Main+O_3	-8.922	-91.682	73.838
LF	PM_{10}_3h	Main+NO_2+O_3	-19.011	-88.873	50.850
LF	PM_{10} 12h	Main+NO_2+O_3	10.233	-78.472	98.938
LF	PM_{10} 24h	Main+NO_2+O_3	12.193	-72.789	97.174
LF	PM_{10} lag0	Main+NO_2+O_3	-5.599	-74.981	63.782
LF	PM_{10} lag1	Main+NO_2+O_3	12.191	-62.061	86.442
LF	PM_{10} lag2	Main+NO_2+O_3	-9.248	-92.225	73.728
HF	PM_{10}_3h	Main, reduced	-50.070	-111.675	11.536
HF	PM_{10} 12h	Main, reduced	-42.079	-128.141	43.983
HF	PM_{10} 24h	Main, reduced	-12.449	-93.856	68.958
HF	PM_{10} lag0	Main, reduced	-35.587	-88.626	17.451
HF	PM_{10} lag1	Main, reduced	2.866	-63.267	69.000
HF	PM_{10} lag2	Main, reduced	7.744	-67.381	82.869
HF	PM_{10}_3h	Main+NO_2	-32.582	-99.431	34.268
HF	PM_{10} 12h	Main+NO_2	-8.875	-104.202	86.452
HF	PM_{10} 24h	Main+NO_2	4.206	-78.755	87.166
HF	PM_{10} lag0	Main+NO_2	-18.831	-76.590	38.928
HF	PM_{10} lag1	Main+NO_2	2.540	-63.246	68.325
HF	PM_{10} lag2	Main+NO_2	2.941	-71.982	77.864
HF	PM_{10}_3h	Main+O_3	-49.974	-112.336	12.389

HF	PM_{10} 12h	Main+O_3	-42.183	-128.445	44.079
HF	PM_{10} 24h	Main+O_3	-14.515	-97.224	68.195
HF	PM_{10} lag0	Main+O_3	-35.315	-88.591	17.960
HF	PM_{10} lag1	Main+O_3	1.682	-65.310	68.673
HF	PM_{10} lag2	Main+O_3	6.507	-69.599	82.612
HF	PM_{10}_3h	Main+NO_2+O_3	-32.999	-100.194	34.195
HF	PM_{10} 12h	Main+NO_2+O_3	-8.552	-104.560	87.456
HF	PM_{10} 24h	Main+NO_2+O_3	5.208	-79.863	90.278
HF	PM_{10} lag0	Main+NO_2+O_3	-18.816	-76.710	39.078
HF	PM_{10} lag1	Main+NO_2+O_3	3.011	-63.636	69.659
HF	PM_{10} lag2	Main+NO_2+O_3	3.440	-72.348	79.227
LF/HF	PM_{10}_3h	Main, reduced	0.026	-0.097	0.149
LF/HF	PM_{10} 12h	Main, reduced	0.019	-0.133	0.172
LF/HF	PM_{10} 24h	Main, reduced	0.003	-0.138	0.144
LF/HF	PM_{10} lag0	Main, reduced	-0.039	-0.149	0.072
LF/HF	PM_{10} lag1	Main, reduced	-0.044	-0.158	0.071
LF/HF	PM_{10} lag2	Main, reduced	-0.115	-0.250	0.021
LF/HF	PM_{10}_3h	Main+NO_2	0.028	-0.150	0.207
LF/HF	PM_{10} 12h	Main+NO_2	-0.013	-0.270	0.244
LF/HF	PM_{10} 24h	Main+NO_2	-0.080	-0.337	0.177
LF/HF	PM_{10} lag0	Main+NO_2	-0.067	-0.216	0.082
LF/HF	PM_{10} lag1	Main+NO_2	-0.175	-0.381	0.031
LF/HF	PM_{10} lag2	Main+NO_2	-0.163	-0.340	0.014
LF/HF	PM_{10}_3h	Main+O_3	0.058	-0.112	0.229
LF/HF	PM_{10} 12h	Main+O_3	0.064	-0.172	0.301
LF/HF	PM_{10} 24h	Main+O_3	0.048	-0.174	0.271
LF/HF	PM_{10} lag0	Main+O_3	-0.056	-0.205	0.093
LF/HF	PM_{10} lag1	Main+O_3	-0.065	-0.252	0.122
LF/HF	PM_{10} lag2	Main+O_3	-0.166	-0.343	0.011
LF/HF	PM_{10}_3h	Main+NO_2+O_3	0.020	-0.160	0.199
LF/HF	PM_{10} 12h	Main+NO_2+O_3	-0.007	-0.264	0.250
LF/HF	PM_{10} 24h	Main+NO_2+O_3	-0.058	-0.321	0.205
LF/HF	PM_{10} lag0	Main+NO_2+O_3	-0.068	-0.217	0.081
LF/HF	PM_{10} lag1	Main+NO_2+O_3	-0.165	-0.373	0.044
LF/HF	PM_{10} lag2	Main+NO_2+O_3	-0.164	-0.340	0.013

Supplementary Figure 5: Change in Outcomes per IQR exposure increase to UFP among Seniors – Sensitivity Analyses Adjusting for Season and Heart Medication

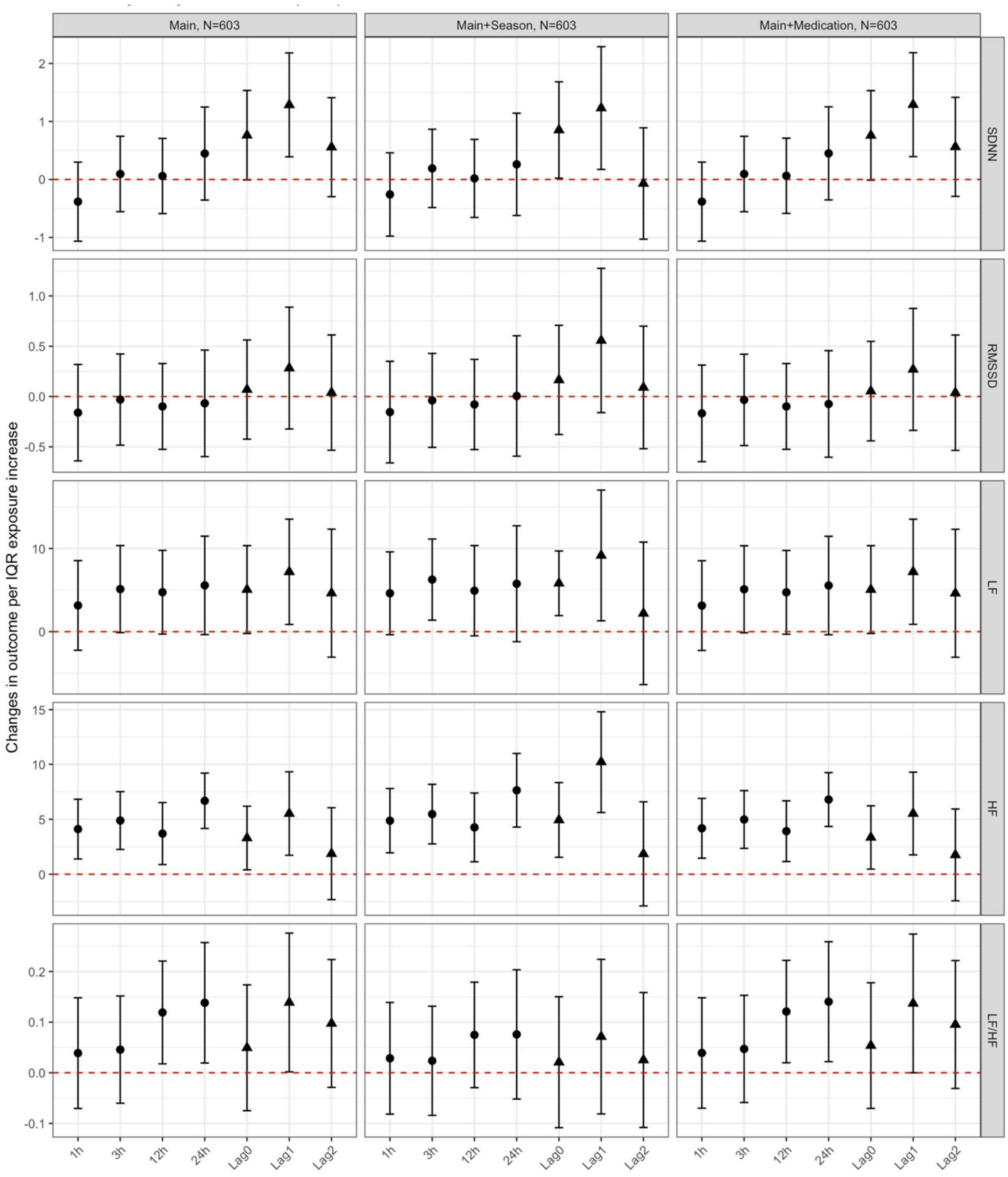

Abbreviations: UFP, ultrafine particles; SDNN, standard deviation of normal to normal intervals; RMSSD, root mean square of successive differences between normal heartbeats; LF, low frequency power; HF, high frequency power; LF/HF, ratio of Low to high frequency power

Supplementary Table 10: Change in Outcomes per IQR exposure increase to UFP among Seniors – Sensitivity Analyses Adjusting for Season and Heart Medication: Estimates and 95% CI

**Outcome**	**Exposure**	**Model**	**Estimate**	**CI low**	**CI high**
SDNN	UFP 1h	Main	-0.383	-1.065	0.298
SDNN	UFP 3h	Main	0.094	-0.555	0.744
SDNN	UFP 12h	Main	0.059	-0.589	0.707
SDNN	UFP 24h	Main	0.447	-0.355	1.249
SDNN	UFP lag0	Main	0.763	-0.009	1.534
SDNN	UFP lag1	Main	1.286	0.390	2.181
SDNN	UFP lag2	Main	0.557	-0.297	1.410
SDNN	UFP 1h	Main+Season	-0.259	-0.978	0.460
SDNN	UFP 3h	Main+Season	0.191	-0.484	0.865
SDNN	UFP 12h	Main+Season	0.018	-0.655	0.691
SDNN	UFP 24h	Main+Season	0.260	-0.621	1.142
SDNN	UFP lag0	Main+Season	0.852	0.021	1.683
SDNN	UFP lag1	Main+Season	1.231	0.173	2.289
SDNN	UFP lag2	Main+Season	-0.070	-1.030	0.890
SDNN	UFP 1h	Main+Medication	-0.383	-1.064	0.299
SDNN	UFP 3h	Main+Medication	0.094	-0.556	0.744
SDNN	UFP 12h	Main+Medication	0.063	-0.585	0.711
SDNN	UFP 24h	Main+Medication	0.449	-0.353	1.252
SDNN	UFP lag0	Main+Medication	0.759	-0.013	1.531
SDNN	UFP lag1	Main+Medication	1.290	0.394	2.185
SDNN	UFP lag2	Main+Medication	0.562	-0.291	1.416
RMSSD	UFP 1h	Main	-0.161	-0.640	0.319
RMSSD	UFP 3h	Main	-0.030	-0.483	0.423
RMSSD	UFP 12h	Main	-0.099	-0.525	0.328
RMSSD	UFP 24h	Main	-0.067	-0.597	0.462
RMSSD	UFP lag0	Main	0.069	-0.424	0.562
RMSSD	UFP lag1	Main	0.283	-0.323	0.888
RMSSD	UFP lag2	Main	0.039	-0.534	0.613
RMSSD	UFP 1h	Main+Season	-0.155	-0.660	0.350
RMSSD	UFP 3h	Main+Season	-0.039	-0.506	0.429
RMSSD	UFP 12h	Main+Season	-0.079	-0.528	0.369
RMSSD	UFP 24h	Main+Season	0.006	-0.593	0.604
RMSSD	UFP lag0	Main+Season	0.165	-0.378	0.708
RMSSD	UFP lag1	Main+Season	0.557	-0.159	1.274
RMSSD	UFP lag2	Main+Season	0.090	-0.519	0.700
RMSSD	UFP 1h	Main+Medication	-0.167	-0.647	0.313
RMSSD	UFP 3h	Main+Medication	-0.033	-0.487	0.420
RMSSD	UFP 12h	Main+Medication	-0.099	-0.525	0.328
RMSSD	UFP 24h	Main+Medication	-0.074	-0.604	0.456
RMSSD	UFP lag0	Main+Medication	0.053	-0.441	0.548

RMSSD	UFP lag1	Main+Medication	0.270	-0.337	0.876
RMSSD	UFP lag2	Main+Medication	0.038	-0.536	0.611
LF	UFP 1h	Main	3.156	-2.238	8.549
LF	UFP 3h	Main	5.120	-0.112	10.353
LF	UFP 12h	Main	4.746	-0.285	9.777
LF	UFP 24h	Main	5.568	-0.345	11.480
LF	UFP lag0	Main	5.063	-0.216	10.342
LF	UFP lag1	Main	7.201	0.868	13.534
LF	UFP lag2	Main	4.633	-3.064	12.330
LF	UFP 1h	Main+Season	4.618	-0.361	9.597
LF	UFP 3h	Main+Season	6.263	1.385	11.142
LF	UFP 12h	Main+Season	4.925	-0.499	10.349
LF	UFP 24h	Main+Season	5.766	-1.203	12.734
LF	UFP lag0	Main+Season	5.821	1.937	9.705
LF	UFP lag1	Main+Season	9.168	1.309	17.028
LF	UFP lag2	Main+Season	2.205	-6.366	10.776
LF	UFP 1h	Main+Medication	3.136	-2.259	8.531
LF	UFP 3h	Main+Medication	5.097	-0.136	10.329
LF	UFP 12h	Main+Medication	4.730	-0.307	9.768
LF	UFP 24h	Main+Medication	5.552	-0.366	11.470
LF	UFP lag0	Main+Medication	5.060	-0.217	10.336
LF	UFP lag1	Main+Medication	7.203	0.880	13.526
LF	UFP lag2	Main+Medication	4.621	-3.073	12.316
HF	UFP 1h	Main	4.106	1.389	6.822
HF	UFP 3h	Main	4.886	2.256	7.516
HF	UFP 12h	Main	3.700	0.881	6.519
HF	UFP 24h	Main	6.678	4.163	9.193
HF	UFP lag0	Main	3.296	0.402	6.190
HF	UFP lag1	Main	5.521	1.725	9.317
HF	UFP lag2	Main	1.873	-2.303	6.049
HF	UFP 1h	Main+Season	4.870	1.947	7.794
HF	UFP 3h	Main+Season	5.467	2.759	8.176
HF	UFP 12h	Main+Season	4.262	1.137	7.388
HF	UFP 24h	Main+Season	7.638	4.289	10.986
HF	UFP lag0	Main+Season	4.938	1.543	8.333
HF	UFP lag1	Main+Season	10.213	5.617	14.810
HF	UFP lag2	Main+Season	1.853	-2.880	6.587
HF	UFP 1h	Main+Medication	4.180	1.461	6.899
HF	UFP 3h	Main+Medication	4.980	2.354	7.605
HF	UFP 12h	Main+Medication	3.918	1.157	6.679
HF	UFP 24h	Main+Medication	6.793	4.342	9.244
HF	UFP lag0	Main+Medication	3.347	0.471	6.224
HF	UFP lag1	Main+Medication	5.524	1.762	9.287
HF	UFP lag2	Main+Medication	1.759	-2.413	5.930

LF/HF	UFP 1h	Main	0.039	-0.070	0.148
LF/HF	UFP 3h	Main	0.046	-0.060	0.152
LF/HF	UFP 12h	Main	0.119	0.018	0.221
LF/HF	UFP 24h	Main	0.138	0.019	0.257
LF/HF	UFP lag0	Main	0.049	-0.075	0.174
LF/HF	UFP lag1	Main	0.139	0.002	0.276
LF/HF	UFP lag2	Main	0.097	-0.029	0.224
LF/HF	UFP 1h	Main+Season	0.029	-0.082	0.139
LF/HF	UFP 3h	Main+Season	0.024	-0.084	0.131
LF/HF	UFP 12h	Main+Season	0.075	-0.029	0.179
LF/HF	UFP 24h	Main+Season	0.076	-0.052	0.204
LF/HF	UFP lag0	Main+Season	0.021	-0.109	0.150
LF/HF	UFP lag1	Main+Season	0.071	-0.081	0.224
LF/HF	UFP lag2	Main+Season	0.025	-0.108	0.159
LF/HF	UFP 1h	Main+Medication	0.039	-0.070	0.148
LF/HF	UFP 3h	Main+Medication	0.047	-0.059	0.153
LF/HF	UFP 12h	Main+Medication	0.121	0.020	0.222
LF/HF	UFP 24h	Main+Medication	0.141	0.022	0.259
LF/HF	UFP lag0	Main+Medication	0.054	-0.070	0.178
LF/HF	UFP lag1	Main+Medication	0.137	0.000	0.274
LF/HF	UFP lag2	Main+Medication	0.095	-0.031	0.222

Supplementary Figure 6: Change in Outcomes per IQR exposure increase to PM_{10} among Seniors – Sensitivity Analyses Adjusting for Season and Heart Medication

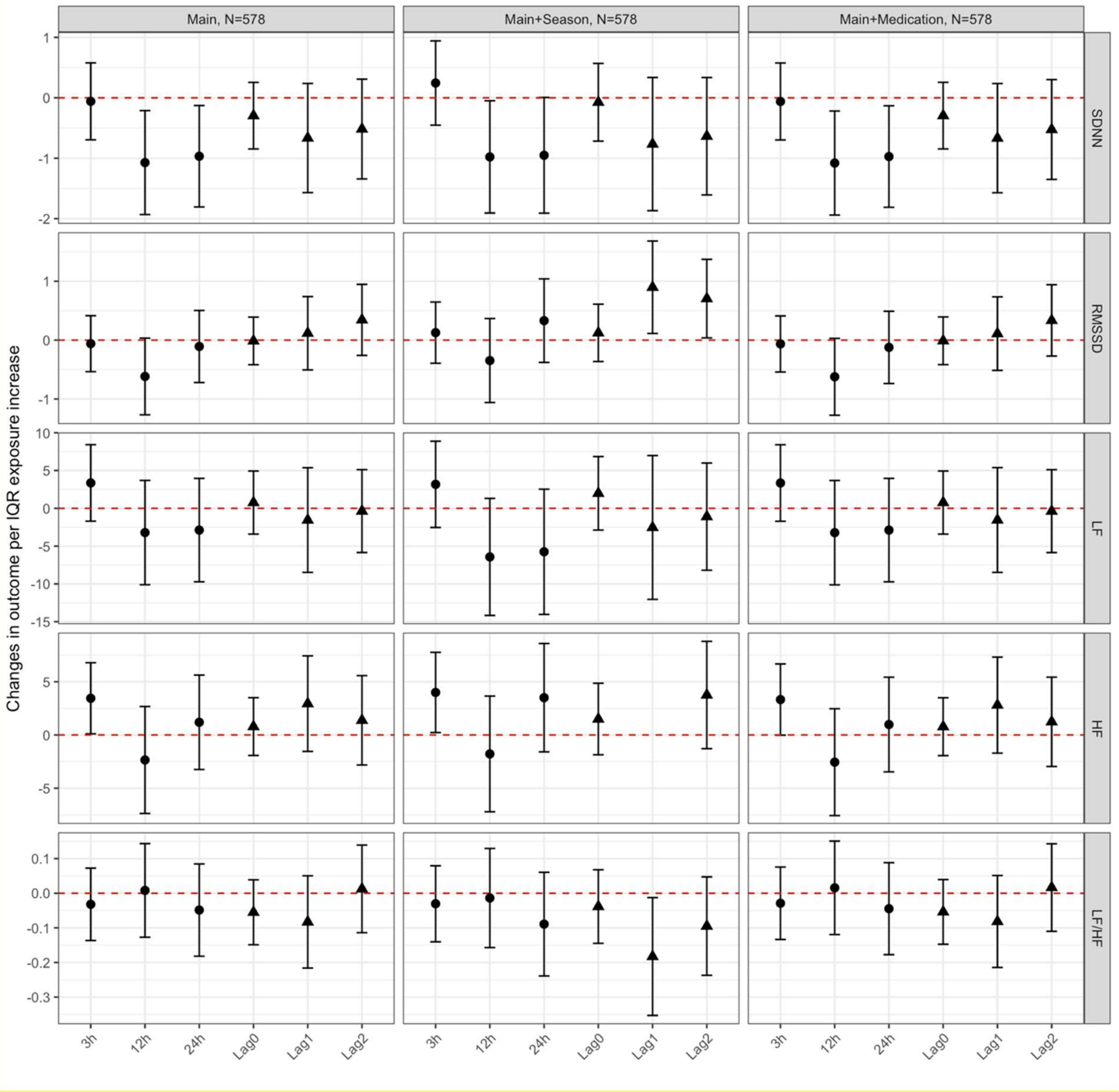

Abbreviations: PM, particulate matter; SDNN, standard deviation of normal to normal intervals; RMSSD, root mean square of successive differences between normal heartbeats; LF, low frequency power; HF, high frequency power; LF/HF, ratio of Low to high frequency power

Supplementary Table 11: Change in Outcomes per IQR exposure increase to PM_{10} among Seniors – Sensitivity Analyses Adjusting for Season and Heart Medication: Estimates and 95% CI

**Outcome**	**Exposure**	**Model**	**Estimate**	**CI low**	**CI high**
SDNN	PM_{10} 3h	Main	-0.059	-0.696	0.578
SDNN	PM_{10} 12h	Main	-1.072	-1.932	-0.212
SDNN	PM_{10} 24h	Main	-0.967	-1.807	-0.128
SDNN	PM_{10}_lag0	Main	-0.296	-0.847	0.255
SDNN	PM_{10} lag1	Main	-0.666	-1.570	0.237
SDNN	PM_{10} lag2	Main	-0.517	-1.343	0.308
SDNN	PM_{10} 3h	Main+Season	0.245	-0.453	0.942
SDNN	PM_{10} 12h	Main+Season	-0.978	-1.907	-0.049
SDNN	PM_{10} 24h	Main+Season	-0.952	-1.910	0.006
SDNN	PM_{10}_lag0	Main+Season	-0.074	-0.717	0.569
SDNN	PM_{10} lag1	Main+Season	-0.766	-1.868	0.336
SDNN	PM_{10} lag2	Main+Season	-0.636	-1.608	0.335
SDNN	PM_{10} 3h	Main+Medication	-0.061	-0.698	0.576
SDNN	PM_{10} 12h	Main+Medication	-1.080	-1.941	-0.219
SDNN	PM_{10} 24h	Main+Medication	-0.973	-1.813	-0.132
SDNN	PM_{10}_lag0	Main+Medication	-0.295	-0.846	0.256
SDNN	PM_{10} lag1	Main+Medication	-0.668	-1.572	0.236
SDNN	PM_{10} lag2	Main+Medication	-0.525	-1.351	0.301
RMSSD	PM_{10} 3h	Main	-0.061	-0.536	0.415
RMSSD	PM_{10} 12h	Main	-0.617	-1.269	0.034
RMSSD	PM_{10} 24h	Main	-0.108	-0.720	0.503
RMSSD	PM_{10}_lag0	Main	-0.014	-0.418	0.391
RMSSD	PM_{10} lag1	Main	0.117	-0.506	0.740
RMSSD	PM_{10} lag2	Main	0.344	-0.260	0.947
RMSSD	PM_{10} 3h	Main+Season	0.127	-0.393	0.646
RMSSD	PM_{10} 12h	Main+Season	-0.347	-1.060	0.366
RMSSD	PM_{10} 24h	Main+Season	0.331	-0.379	1.040
RMSSD	PM_{10}_lag0	Main+Season	0.123	-0.363	0.610
RMSSD	PM_{10} lag1	Main+Season	0.898	0.113	1.682
RMSSD	PM_{10} lag2	Main+Season	0.705	0.038	1.371
RMSSD	PM_{10} 3h	Main+Medication	-0.065	-0.541	0.411
RMSSD	PM_{10} 12h	Main+Medication	-0.623	-1.276	0.030
RMSSD	PM_{10} 24h	Main+Medication	-0.123	-0.737	0.491
RMSSD	PM_{10}_lag0	Main+Medication	-0.012	-0.417	0.394
RMSSD	PM_{10} lag1	Main+Medication	0.110	-0.514	0.734
RMSSD	PM_{10} lag2	Main+Medication	0.334	-0.271	0.940
LF	PM_{10} 3h	Main	3.360	-1.699	8.419
LF	PM_{10} 12h	Main	-3.209	-10.112	3.693
LF	PM_{10} 24h	Main	-2.879	-9.719	3.961
LF	PM_{10}_lag0	Main	0.760	-3.411	4.932

LF	PM_{10} lag1	Main	-1.553	-8.477	5.371
LF	PM_{10} lag2	Main	-0.366	-5.848	5.116
LF	PM_{10} 3h	Main+Season	3.171	-2.538	8.879
LF	PM_{10} 12h	Main+Season	-6.428	-14.169	1.314
LF	PM_{10} 24h	Main+Season	-5.750	-14.033	2.534
LF	PM_{10}_lag0	Main+Season	1.982	-2.880	6.844
LF	PM_{10} lag1	Main+Season	-2.535	-12.049	6.979
LF	PM_{10} lag2	Main+Season	-1.099	-8.189	5.992
LF	PM_{10} 3h	Main+Medication	3.348	-1.715	8.412
LF	PM_{10} 12h	Main+Medication	-3.222	-10.127	3.683
LF	PM_{10} 24h	Main+Medication	-2.881	-9.723	3.961
LF	PM_{10}_lag0	Main+Medication	0.761	-3.413	4.934
LF	PM_{10} lag1	Main+Medication	-1.546	-8.476	5.384
LF	PM_{10} lag2	Main+Medication	-0.372	-5.856	5.112
HF	PM_{10} 3h	Main	3.448	0.107	6.790
HF	PM_{10} 12h	Main	-2.348	-7.372	2.677
HF	PM_{10} 24h	Main	1.193	-3.243	5.628
HF	PM_{10}_lag0	Main	0.788	-1.927	3.502
HF	PM_{10} lag1	Main	2.943	-1.545	7.431
HF	PM_{10} lag2	Main	1.380	-2.820	5.579
HF	PM_{10} 3h	Main+Season	3.998	0.229	7.766
HF	PM_{10} 12h	Main+Season	-1.780	-7.216	3.656
HF	PM_{10} 24h	Main+Season	3.504	-1.586	8.595
HF	PM_{10}_lag0	Main+Season	1.500	-1.861	4.861
HF	PM_{10} lag1	Main+Season	8.914	-17.425	35.252
HF	PM_{10} lag2	Main+Season	3.755	-1.286	8.795
HF	PM_{10} 3h	Main+Medication	3.327	-0.019	6.673
HF	PM_{10} 12h	Main+Medication	-2.555	-7.575	2.466
HF	PM_{10} 24h	Main+Medication	0.982	-3.465	5.429
HF	PM_{10}_lag0	Main+Medication	0.777	-1.942	3.496
HF	PM_{10} lag1	Main+Medication	2.810	-1.704	7.324
HF	PM_{10} lag2	Main+Medication	1.237	-2.961	5.434
LF/HF	PM_{10} 3h	Main	-0.032	-0.137	0.073
LF/HF	PM_{10} 12h	Main	0.008	-0.127	0.143
LF/HF	PM_{10} 24h	Main	-0.049	-0.182	0.085
LF/HF	PM_{10}_lag0	Main	-0.055	-0.149	0.039
LF/HF	PM_{10} lag1	Main	-0.083	-0.216	0.050
LF/HF	PM_{10} lag2	Main	0.013	-0.114	0.139
LF/HF	PM_{10} 3h	Main+Season	-0.030	-0.140	0.079
LF/HF	PM_{10} 12h	Main+Season	-0.014	-0.157	0.129
LF/HF	PM_{10} 24h	Main+Season	-0.089	-0.239	0.061
LF/HF	PM_{10}_lag0	Main+Season	-0.038	-0.145	0.068
LF/HF	PM_{10} lag1	Main+Season	-0.183	-0.353	-0.013
LF/HF	PM_{10} lag2	Main+Season	-0.095	-0.237	0.047

LF/HF	PM_{10} 3h	Main+Medication	-0.029	-0.133	0.076
LF/HF	PM_{10} 12h	Main+Medication	0.016	-0.119	0.151
LF/HF	PM_{10} 24h	Main+Medication	-0.045	-0.177	0.088
LF/HF	PM_{10}_lag0	Main+Medication	-0.054	-0.147	0.039
LF/HF	PM_{10} lag1	Main+Medication	-0.082	-0.214	0.051
LF/HF	PM_{10} lag2	Main+Medication	0.016	-0.110	0.143

Supplementary Figure 7: Change in Outcomes per IQR exposure increase to UFP among Children – Sensitivity Analyses Adjusting for Season and Hay Fever

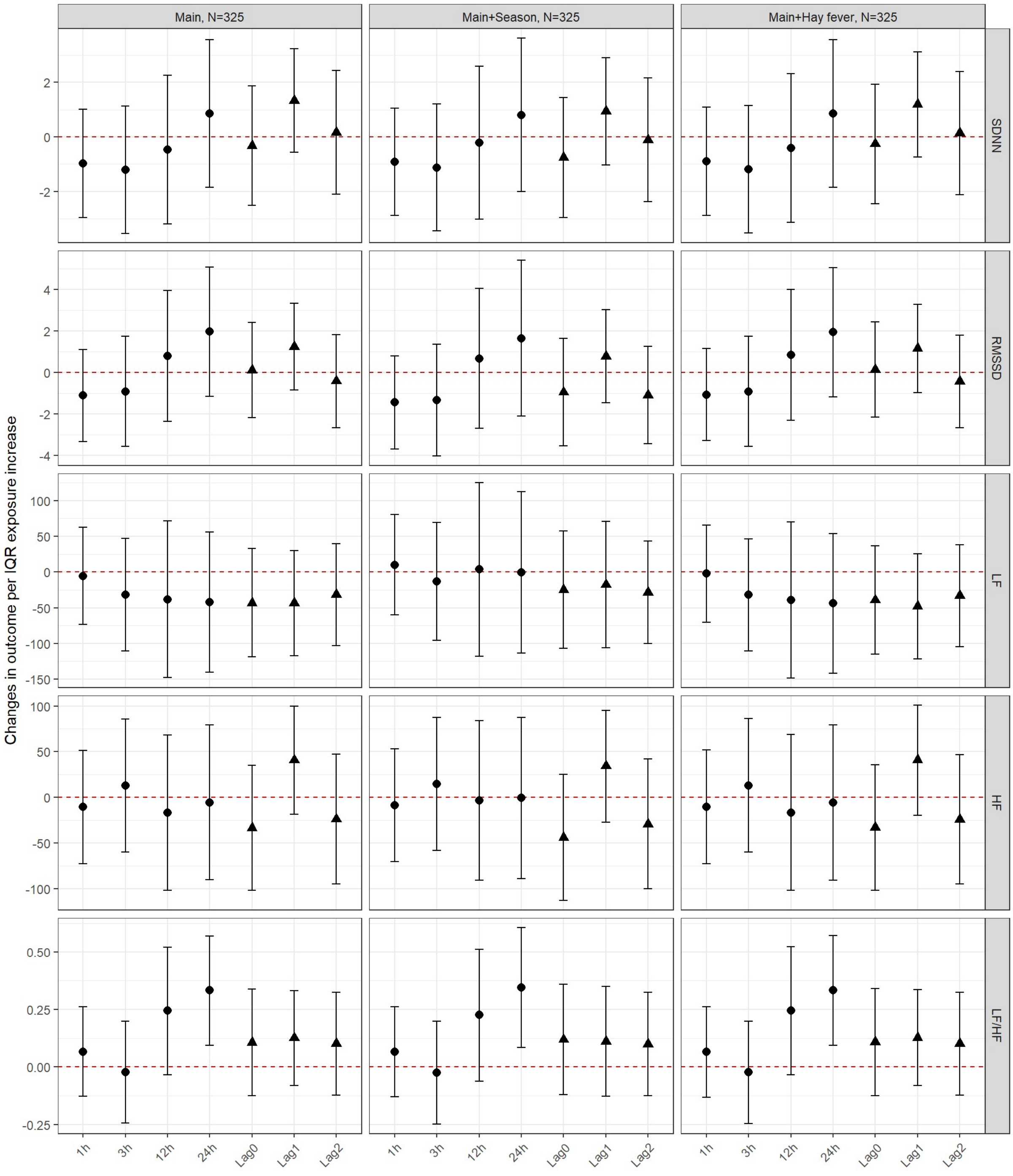

Abbreviations: UFP, ultrafine particles; SDNN, standard deviation of normal to normal intervals; RMSSD, root mean square of successive differences between normal heartbeats; LF, low frequency power; HF, high frequency power; LF/HF, ratio of Low to high frequency power

Supplementary Table 12: Change in Outcomes per IQR exposure increase to UFP among Children – Sensitivity Analyses Adjusting for Season and Hay Fever: Estimates and 95% CI

Outcome	Exposure	Model	Estimate	CI low	CI high
SDNN	UFP 1h	Main	-0.966	-2.948	1.017
SDNN	UFP 3h	Main	-1.207	-3.541	1.128
SDNN	UFP 12h	Main	-0.472	-3.192	2.247
SDNN	UFP 24h	Main	0.854	-1.849	3.556
SDNN	UFP lag0	Main	-0.326	-2.510	1.857
SDNN	UFP lag1	Main	1.331	-0.565	3.228
SDNN	UFP lag2	Main	0.164	-2.097	2.424
SDNN	UFP 1h	Main+Season	-0.915	-2.888	1.057
SDNN	UFP 3h	Main+Season	-1.126	-3.449	1.197
SDNN	UFP 12h	Main+Season	-0.215	-3.007	2.577
SDNN	UFP 24h	Main+Season	0.801	-2.006	3.607
SDNN	UFP lag0	Main+Season	-0.755	-2.958	1.447
SDNN	UFP lag1	Main+Season	0.936	-1.024	2.895
SDNN	UFP lag2	Main+Season	-0.104	-2.371	2.162
SDNN	UFP 1h	Main+Hay fever	-0.890	-2.878	1.098
SDNN	UFP 3h	Main+Hay fever	-1.181	-3.516	1.154
SDNN	UFP 12h	Main+Hay fever	-0.404	-3.126	2.318
SDNN	UFP 24h	Main+Hay fever	0.853	-1.849	3.555
SDNN	UFP lag0	Main+Hay fever	-0.256	-2.443	1.931
SDNN	UFP lag1	Main+Hay fever	1.188	-0.738	3.114
SDNN	UFP lag2	Main+Hay fever	0.135	-2.126	2.396
RMSSD	UFP 1h	Main	-1.110	-3.324	1.104
RMSSD	UFP 3h	Main	-0.907	-3.560	1.747
RMSSD	UFP 12h	Main	0.810	-2.344	3.963
RMSSD	UFP 24h	Main	1.973	-1.148	5.093
RMSSD	UFP lag0	Main	0.110	-2.188	2.408
RMSSD	UFP lag1	Main	1.255	-0.839	3.349
RMSSD	UFP lag2	Main	-0.410	-2.653	1.833
RMSSD	UFP 1h	Main+Season	-1.441	-3.683	0.802
RMSSD	UFP 3h	Main+Season	-1.334	-4.023	1.356
RMSSD	UFP 12h	Main+Season	0.682	-2.696	4.061
RMSSD	UFP 24h	Main+Season	1.654	-2.105	5.413
RMSSD	UFP lag0	Main+Season	-0.947	-3.532	1.639
RMSSD	UFP lag1	Main+Season	0.785	-1.461	3.032
RMSSD	UFP lag2	Main+Season	-1.085	-3.439	1.268
RMSSD	UFP 1h	Main+Hay fever	-1.067	-3.287	1.153
RMSSD	UFP 3h	Main+Hay fever	-0.908	-3.564	1.748
RMSSD	UFP 12h	Main+Hay fever	0.844	-2.313	4.002
RMSSD	UFP 24h	Main+Hay fever	1.949	-1.175	5.073
RMSSD	UFP lag0	Main+Hay fever	0.144	-2.158	2.447
RMSSD	UFP lag1	Main+Hay fever	1.163	-0.960	3.287

RMSSD	UFP lag2	Main+Hay fever	-0.429	-2.675	1.816
LF	UFP 1h	Main	-5.408	-73.579	62.763
LF	UFP 3h	Main	-31.742	-110.235	46.752
LF	UFP 12h	Main	-38.144	-147.690	71.402
LF	UFP 24h	Main	-42.359	-140.391	55.672
LF	UFP lag0	Main	-43.078	-118.901	32.744
LF	UFP lag1	Main	-43.627	-117.256	30.002
LF	UFP lag2	Main	-31.551	-103.103	40.000
LF	UFP 1h	Main+Season	10.068	-60.321	80.456
LF	UFP 3h	Main+Season	-12.871	-95.464	69.721
LF	UFP 12h	Main+Season	3.606	-117.729	124.940
LF	UFP 24h	Main+Season	-0.156	-113.189	112.876
LF	UFP lag0	Main+Season	-24.842	-107.203	57.519
LF	UFP lag1	Main+Season	-17.660	-106.082	70.762
LF	UFP lag2	Main+Season	-28.207	-99.956	43.542
LF	UFP 1h	Main+Hay fever	-2.086	-70.210	66.038
LF	UFP 3h	Main+Hay fever	-31.947	-110.217	46.324
LF	UFP 12h	Main+Hay fever	-39.189	-148.414	70.035
LF	UFP 24h	Main+Hay fever	-43.698	-141.446	54.049
LF	UFP lag0	Main+Hay fever	-39.208	-115.014	36.599
LF	UFP lag1	Main+Hay fever	-47.979	-121.539	25.581
LF	UFP lag2	Main+Hay fever	-33.055	-104.409	38.298
HF	UFP 1h	Main	-10.623	-72.570	51.325
HF	UFP 3h	Main	12.884	-59.882	85.651
HF	UFP 12h	Main	-16.925	-101.851	68.001
HF	UFP 24h	Main	-5.585	-90.137	78.968
HF	UFP lag0	Main	-33.359	-101.519	34.801
HF	UFP lag1	Main	40.685	-18.563	99.933
HF	UFP lag2	Main	-23.787	-94.420	46.846
HF	UFP 1h	Main+Season	-8.670	-70.410	53.070
HF	UFP 3h	Main+Season	14.551	-57.984	87.087
HF	UFP 12h	Main+Season	-3.365	-90.732	84.003
HF	UFP 24h	Main+Season	-0.702	-88.652	87.247
HF	UFP lag0	Main+Season	-43.662	-112.509	25.186
HF	UFP lag1	Main+Season	34.218	-27.083	95.519
HF	UFP lag2	Main+Season	-29.178	-100.099	41.743
HF	UFP 1h	Main+Hay fever	-10.166	-72.372	52.040
HF	UFP 3h	Main+Hay fever	13.072	-59.829	85.973
HF	UFP 12h	Main+Hay fever	-16.508	-101.677	68.660
HF	UFP 24h	Main+Hay fever	-5.578	-90.281	79.125
HF	UFP lag0	Main+Hay fever	-33.022	-101.423	35.378
HF	UFP lag1	Main+Hay fever	40.738	-19.525	101.002
HF	UFP lag2	Main+Hay fever	-24.008	-94.787	46.771
LF/HF	UFP 1h	Main	0.066	-0.128	0.261

LF/HF	UFP 3h	Main	-0.022	-0.244	0.199
LF/HF	UFP 12h	Main	0.244	-0.033	0.521
LF/HF	UFP 24h	Main	0.332	0.093	0.570
LF/HF	UFP lag0	Main	0.106	-0.125	0.337
LF/HF	UFP lag1	Main	0.126	-0.080	0.331
LF/HF	UFP lag2	Main	0.101	-0.121	0.322
LF/HF	UFP 1h	Main+Season	0.066	-0.129	0.261
LF/HF	UFP 3h	Main+Season	-0.025	-0.247	0.197
LF/HF	UFP 12h	Main+Season	0.226	-0.061	0.512
LF/HF	UFP 24h	Main+Season	0.345	0.084	0.606
LF/HF	UFP lag0	Main+Season	0.119	-0.119	0.357
LF/HF	UFP lag1	Main+Season	0.111	-0.126	0.348
LF/HF	UFP lag2	Main+Season	0.100	-0.125	0.324
LF/HF	UFP 1h	Main+Hay fever	0.066	-0.130	0.262
LF/HF	UFP 3h	Main+Hay fever	-0.022	-0.244	0.199
LF/HF	UFP 12h	Main+Hay fever	0.245	-0.034	0.523
LF/HF	UFP 24h	Main+Hay fever	0.333	0.093	0.572
LF/HF	UFP lag0	Main+Hay fever	0.108	-0.125	0.340
LF/HF	UFP lag1	Main+Hay fever	0.128	-0.079	0.334
LF/HF	UFP lag2	Main+Hay fever	0.101	-0.121	0.323

Supplementary Figure 8: Change in Outcomes per IQR exposure increase to PM_{10} among Children – Sensitivity Analyses Adjusting for Season and Hay Fever

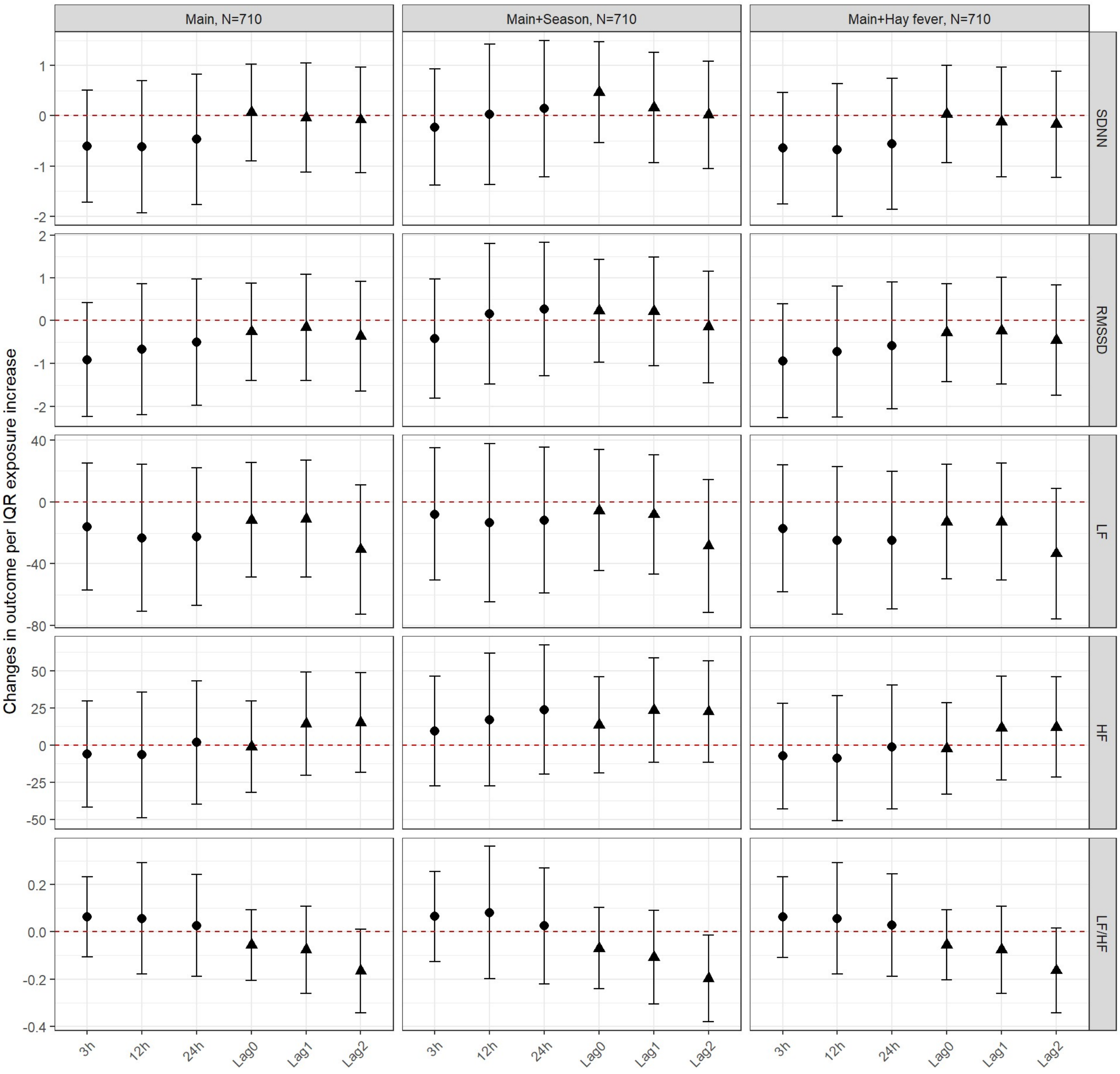

Abbreviations: PM, particulate matter; SDNN, standard deviation of normal to normal intervals; RMSSD, root mean square of successive differences between normal heartbeats; LF, low frequency power; HF, high frequency power; LF/HF, ratio of Low to high frequency power

Supplementary Table 13: Change in Outcomes per IQR exposure increase to PM_{10} among Children – Sensitivity Analyses Adjusting for Season and Hay Fever: Estimates and 95% CI

Outcome	Exposure	Model	Estimate	CI low	CI high
SDNN	PM_{10} 3h	Main	-0.608	-1.721	0.505
SDNN	PM_{10} 12h	Main	-0.619	-1.936	0.698
SDNN	PM_{10} 24h	Main	-0.467	-1.765	0.831
SDNN	PM_{10} lag0	Main	0.067	-0.897	1.030
SDNN	PM_{10} lag1	Main	-0.036	-1.119	1.047
SDNN	PM_{10} lag2	Main	-0.079	-1.128	0.970
SDNN	PM_{10} 3h	Main+Season	-0.226	-1.378	0.927
SDNN	PM_{10} 12h	Main+Season	0.028	-1.367	1.423
SDNN	PM_{10} 24h	Main+Season	0.142	-1.213	1.496
SDNN	PM_{10} lag0	Main+Season	0.470	-0.538	1.477
SDNN	PM_{10} lag1	Main+Season	0.162	-0.935	1.259
SDNN	PM_{10} lag2	Main+Season	0.017	-1.046	1.081
SDNN	PM_{10} 3h	Main+Hay fever	-0.645	-1.759	0.469
SDNN	PM_{10} 12h	Main+Hay fever	-0.679	-1.998	0.641
SDNN	PM_{10} 24h	Main+Hay fever	-0.558	-1.862	0.747
SDNN	PM_{10} lag0	Main+Hay fever	0.036	-0.929	1.000
SDNN	PM_{10} lag1	Main+Hay fever	-0.120	-1.211	0.971
SDNN	PM_{10} lag2	Main+Hay fever	-0.171	-1.229	0.888
RMSSD	PM_{10} 3h	Main	-0.911	-2.233	0.412
RMSSD	PM_{10} 12h	Main	-0.668	-2.193	0.856
RMSSD	PM_{10} 24h	Main	-0.504	-1.976	0.969
RMSSD	PM_{10} lag0	Main	-0.258	-1.392	0.877
RMSSD	PM_{10} lag1	Main	-0.158	-1.400	1.083
RMSSD	PM_{10} lag2	Main	-0.365	-1.641	0.912
RMSSD	PM_{10} 3h	Main+Season	-0.419	-1.809	0.971
RMSSD	PM_{10} 12h	Main+Season	0.162	-1.482	1.807
RMSSD	PM_{10} 24h	Main+Season	0.273	-1.287	1.833
RMSSD	PM_{10} lag0	Main+Season	0.231	-0.972	1.434
RMSSD	PM_{10} lag1	Main+Season	0.215	-1.057	1.486
RMSSD	PM_{10} lag2	Main+Season	-0.142	-1.448	1.165
RMSSD	PM_{10} 3h	Main+Hay fever	-0.936	-2.259	0.386
RMSSD	PM_{10} 12h	Main+Hay fever	-0.716	-2.241	0.810
RMSSD	PM_{10} 24h	Main+Hay fever	-0.580	-2.058	0.897
RMSSD	PM_{10} lag0	Main+Hay fever	-0.281	-1.416	0.853
RMSSD	PM_{10} lag1	Main+Hay fever	-0.234	-1.481	1.013
RMSSD	PM_{10} lag2	Main+Hay fever	-0.459	-1.744	0.825
LF	PM_{10} 3h	Main	-15.926	-56.974	25.121
LF	PM_{10} 12h	Main	-23.202	-70.886	24.483
LF	PM_{10} 24h	Main	-22.509	-66.942	21.924
LF	PM_{10} lag0	Main	-11.598	-48.678	25.482

LF	PM_{10} lag1	Main	-10.867	-48.601	26.866
LF	PM_{10} lag2	Main	-30.681	-72.580	11.217
LF	PM_{10} 3h	Main+Season	-7.848	-50.556	34.859
LF	PM_{10} 12h	Main+Season	-13.486	-64.573	37.600
LF	PM_{10} 24h	Main+Season	-11.778	-59.014	35.458
LF	PM_{10} lag0	Main+Season	-5.434	-44.732	33.864
LF	PM_{10} lag1	Main+Season	-8.047	-46.688	30.594
LF	PM_{10} lag2	Main+Season	-28.411	-71.416	14.595
LF	PM_{10} 3h	Main+Hay fever	-17.024	-58.133	24.084
LF	PM_{10} 12h	Main+Hay fever	-24.805	-72.595	22.985
LF	PM_{10} 24h	Main+Hay fever	-24.826	-69.477	19.824
LF	PM_{10} lag0	Main+Hay fever	-12.745	-49.900	24.410
LF	PM_{10} lag1	Main+Hay fever	-12.874	-50.818	25.071
LF	PM_{10} lag2	Main+Hay fever	-33.572	-75.774	8.629
HF	PM_{10} 3h	Main	-5.990	-41.513	29.533
HF	PM_{10} 12h	Main	-6.591	-48.619	35.437
HF	PM_{10} 24h	Main	1.808	-39.610	43.226
HF	PM_{10} lag0	Main	-0.979	-31.715	29.758
HF	PM_{10} lag1	Main	14.372	-20.127	48.870
HF	PM_{10} lag2	Main	15.166	-18.252	48.585
HF	PM_{10} 3h	Main+Season	9.382	-27.536	46.300
HF	PM_{10} 12h	Main+Season	17.148	-27.535	61.832
HF	PM_{10} 24h	Main+Season	23.801	-19.539	67.141
HF	PM_{10} lag0	Main+Season	13.625	-18.655	45.905
HF	PM_{10} lag1	Main+Season	23.490	-11.591	58.572
HF	PM_{10} lag2	Main+Season	22.490	-11.520	56.499
HF	PM_{10} 3h	Main+Hay fever	-7.342	-42.881	28.197
HF	PM_{10} 12h	Main+Hay fever	-8.738	-50.828	33.352
HF	PM_{10} 24h	Main+Hay fever	-1.369	-42.983	40.245
HF	PM_{10} lag0	Main+Hay fever	-2.155	-32.908	28.598
HF	PM_{10} lag1	Main+Hay fever	11.464	-23.276	46.205
HF	PM_{10} lag2	Main+Hay fever	12.059	-21.660	45.777
LF/HF	PM_{10} 3h	Main	0.062	-0.107	0.232
LF/HF	PM_{10} 12h	Main	0.056	-0.180	0.291
LF/HF	PM_{10} 24h	Main	0.027	-0.189	0.243
LF/HF	PM_{10} lag0	Main	-0.056	-0.025	0.093
LF/HF	PM_{10} lag1	Main	-0.076	-0.259	0.107
LF/HF	PM_{10} lag2	Main	-0.166	-0.342	0.011
LF/HF	PM_{10} 3h	Main+Season	0.064	-0.126	0.254
LF/HF	PM_{10} 12h	Main+Season	0.080	-0.199	0.360
LF/HF	PM_{10} 24h	Main+Season	0.025	-0.219	0.268
LF/HF	PM_{10} lag0	Main+Season	-0.070	-0.242	0.101
LF/HF	PM_{10} lag1	Main+Season	-0.108	-0.306	0.089
LF/HF	PM_{10} lag2	Main+Season	-0.197	-0.380	-0.014

LF/HF	PM_{10} 3h	Main+Hay fever	0.062	-0.108	0.232
LF/HF	PM_{10} 12h	Main+Hay fever	0.056	-0.179	0.292
LF/HF	PM_{10} 24h	Main+Hay fever	0.028	-0.189	0.245
LF/HF	PM_{10} lag0	Main+Hay fever	-0.056	-0.204	0.093
LF/HF	PM_{10} lag1	Main+Hay fever	-0.076	-0.260	0.108
LF/HF	PM_{10} lag2	Main+Hay fever	-0.164	-0.342	0.015

Abbreviations: PM, particulate matter; SDNN, standard deviation of normal to normal intervals; RMSSD, root mean square of successive differences between normal heartbeats; LF, low frequency power; HF, high frequency power; LF/HF, ratio of Low to high frequency power

Supplementary Figure 9: Change in Outcomes per IQR exposure increase to UFP among Seniors – Effect Modification by Hypertension

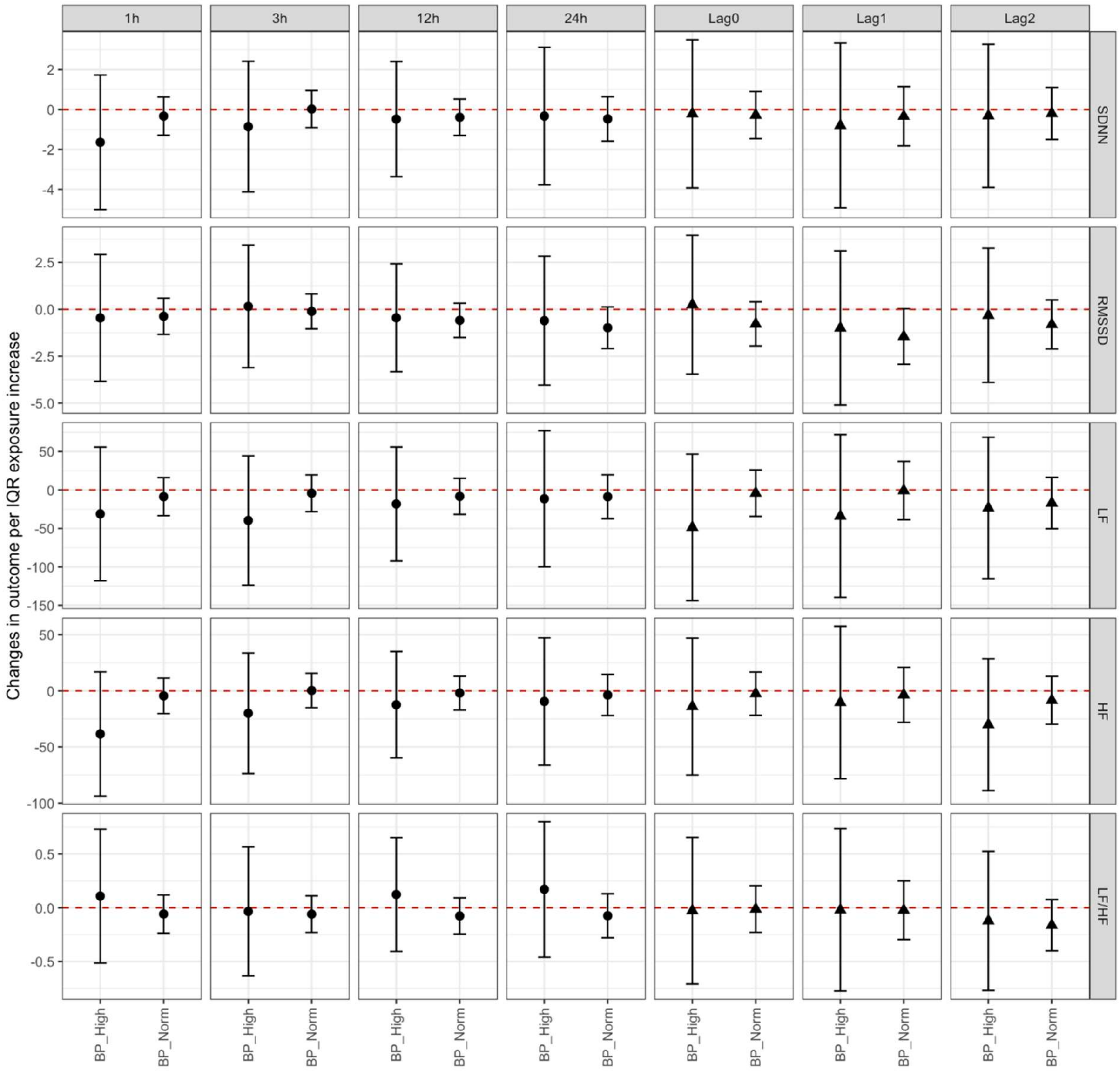

Abbreviations: UFP, ultrafine particles; SDNN, standard deviation of normal to normal intervals; RMSSD, root mean square of successive differences between normal heartbeats; LF, low frequency power; HF, high frequency power; LF/HF, ratio of Low to high frequency power

Supplementary Figure 10: Change in Outcomes per IQR exposure increase to PM10 among Seniors – Effect Modification by Hypertension

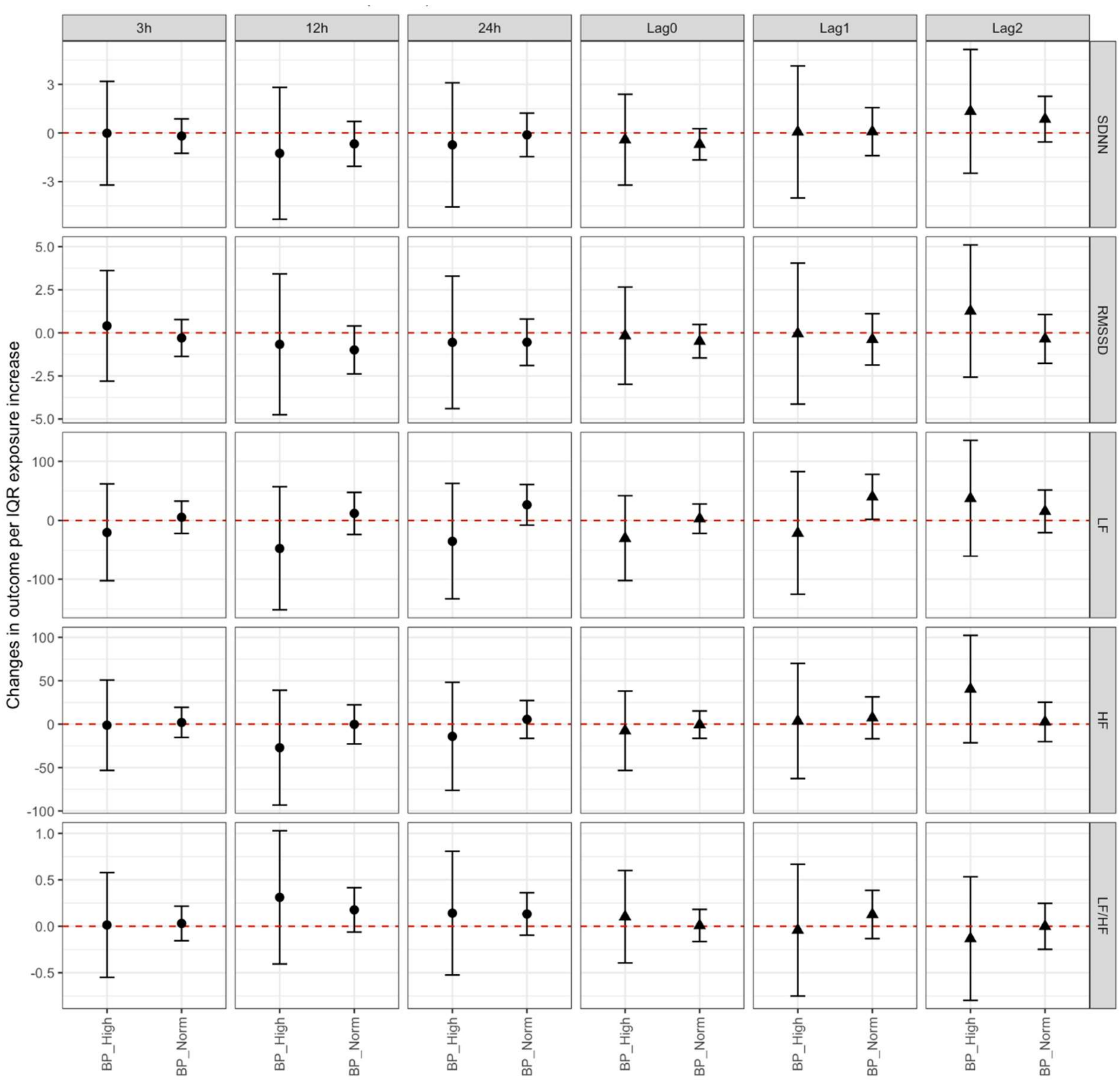

Abbreviations: PM, particulate matter; SDNN, standard deviation of normal to normal intervals; RMSSD, root mean square of successive differences between normal heartbeats; LF, low frequency power; HF, high frequency power; LF/HF, ratio of Low to high frequency power
